# The long non-coding RNA landscape of *Candida* yeast pathogens

**DOI:** 10.1038/s41467-021-27635-4

**Published:** 2021-12-16

**Authors:** Hrant Hovhannisyan, Toni Gabaldón

**Affiliations:** 1 Life Sciences Department, Barcelona Supercomputing Center (BSC), Jordi Girona, 29, 08034 Barcelona, Spain; 2 Mechanisms of Disease Department, Institute for Research in Biomedicine (IRB), Carrer de Baldiri Reixac 10, 08028 Barcelona, Spain; 3 Universitat Pompeu Fabra (UPF), 08003 Barcelona, Spain; 4 Institució Catalana de Recerca i Estudis Avançats (ICREA), Passeig Lluís Companys 23, 08010 Barcelona, Spain; 5 Centro de Investigación Biomédica en Red de Enfermedades Infecciosas, Barcelona, Spain

**Keywords:** Fungal genomics, Fungal pathogenesis, Long non-coding RNAs, Pathogens

## Abstract

Long non-coding RNAs (lncRNAs) constitute a poorly studied class of transcripts with emerging roles in key cellular processes. Despite efforts to characterize lncRNAs across a wide range of species, these molecules remain largely unexplored in most eukaryotic microbes, including yeast pathogens of the *Candida* clade. Here, we analyze thousands of publicly available sequencing datasets to infer and characterize the lncRNA repertoires of five major *Candida* pathogens: *Candida albicans, Candida tropicalis, Candida parapsilosis, Candida auris* and *Candida glabrata*. Our results indicate that genomes of these species encode hundreds of lncRNAs that show levels of evolutionary constraint intermediate between those of intergenic genomic regions and protein-coding genes. Despite their low sequence conservation across the studied species, some lncRNAs are syntenic and are enriched in shared sequence motifs. We find co-expression of lncRNAs with certain protein-coding transcripts, hinting at potential functional associations. Finally, we identify lncRNAs that are differentially expressed during infection of human epithelial cells for four of the studied species. Our comprehensive bioinformatic analyses of *Candida* lncRNAs pave the way for future functional characterization of these transcripts.

## Abstract (web summary)

Long non-coding RNAs (lncRNAs) play roles in key cellular processes, but remain largely unexplored in fungal pathogens such as *Candida*. Here, Hovhannisyan and Gabaldón analyze thousands of sequencing datasets to infer and characterize the lncRNA repertoires of five *Candida* species, paving the way for their future functional characterization.

## Introduction

Advances in high-throughput RNA sequencing (RNA-Seq) in the past decade have shown that eukaryotic cells express abundant and numerous types of non-coding transcripts^1–6^. One major type of non-coding transcripts is long non-coding RNAs (lncRNAs), which are broadly defined as transcripts longer than 200 bp which do not code for proteins. These molecules have several peculiarities that are consistently reported across a wide range of taxa, including mammals, insects, and plants. Firstly, as compared to protein-coding genes they are generally expressed at lower levels and with a higher cell type specificity^7–9^. Secondly, they are poorly conserved at the sequence level, and show a rapid evolutionary turnover, being often species-specific^10–14^. It has been suggested that the functionality of lncRNAs can be attributed to their secondary structure^15–17^, which can be maintained by selective pressures^18^. However, whether lncRNAs are highly structured is still debated^19,20^. LncRNAs have been shown to play important roles in numerous processes such as, among others, gene expression regulation, imprinting, splicing, and cell cycle^21–23^. However, despite extensive research, only a limited number of lncRNAs has been functionally characterized, such as Xist^24^, HOTAIR^25^, Malat1^26^, NORAD^27^, ASCO^28^, among others (see^29^ for an extensive review).

Non-coding transcripts are also abundant in fungi where they have been shown to regulate various processes including cell wall remodeling, transcriptional control, and response to nutrients^6,30–32^. Most of our knowledge about fungal lncRNAs comes from the model organisms *Saccharomyces cerevisiae*^5,32–36^ and *Schizosaccharomyces pombe*^37–40^ but research on lncRNAs has recently expanded to other fungi, such as *Neurospora crassa*^41^*, Fusarium graminearum*^42^*, Metarhizium robertsii*^43^*, Pichia pastoris*^44^, the white-rot fungus *Ganoderma lucidum*^45^, and the brown-rot fungi *Coniophora puteana* and *Serpula lacrymans*^46^. In accordance with findings for other taxa^47,48^, studies on fungi consistently show that lncRNAs are generally shorter, have lower levels of expression and GC content, as compared to protein-coding genes. Some of these studies suggested novel putative functional implications of lncRNAs. For example, one study^43^ identified 1081 lncRNA transcripts in the filamentous fungus *M. robetsii* that were differentially expressed upon heat shock, hinting at a potential implication in thermal stress response. In *F. graminearum* the expression of numerous lncRNAs was shown to be regulated in a stage-specific manner during fruiting body development^42^.

A very limited number of studies, however, have been performed to characterize the role of lncRNAs in fungal virulence. Wang and colleagues^49^ investigated these transcripts in the insect pathogen *Cordyceps militaris*, where >4000 lncRNAs were shown to be regulated during development. Moreover, when *xrn1*, the final gene of the nonsense-mediated decay pathway, determining the fate of lncRNAs, was knocked-out, an attenuation of virulence and growth rate was observed. The role of lncRNAs in fungal pathogenicity towards humans has been studied in the basidiomycete *Cryptococcus neoformans*. Chacko et al.^50^ identified a genomic locus called *RZE1* and found that deletion of this locus results in non-filamentous phenotypes, while its reintroduction restores filamentation. Using site-directed mutagenesis to alter potential translation start codons of *RZE1*, the authors have shown that this locus can control filamentation even with disrupted putative start codons, indicating that *RZE1* functions as a lncRNA, which was also supported by very low sequence conservation of this region across the *Cryptococcus* clade. Further experiments indicated that *RZE1* functions within the nucleus and supposedly indirectly modulates the expression and export of the Znf2 transcription factor which controls morphotype transition in *Cr. neoformans*. Interestingly, *RZE1* and *ZNF2* have proximal genomic positions, and the location of *RZE1* rather than its expression per se had a stronger effect on the expression of *ZNF2*. This was the first study providing evidence of the involvement of lncRNAs in fungal pathogenicity. However, the possible implications of lncRNAs in fungal virulence in other major fungal pathogens remain unknown.

Yeasts from the non-monophyletic *Candida* genus are among the most widespread human fungal opportunistic pathogens. Up to 30 distinct, phylogenetically diverse, *Candida* species have been reported to infect humans, mainly when immunocompromised^51,52^. *Candida* infections represent a high burden for global healthcare. They range from common superficial infections, such as vaginal candidiasis affecting 75% of the female population^53^, to life-threatening invasive infection, with mortality rates reaching 70%^54,55^. Most *Candida* infections are caused by four species, namely *Candida albicans*, *Candida glabrata*, *Candida parapsilosis*, and *Candida tropicalis*, which together account for 85–90% of the cases^56^. In addition, *Candida auris* has recently emerged as a multidrug-resistant yeast pathogen causing numerous hospital-related deadly outbreaks across the globe^57^. Numerous studies have investigated interactions between *Candida* pathogens and different hosts at the level of gene expression by using various techniques, such as microarrays^58–60^ and transcriptome sequencing^61^. However, for the fungal side, all these studies are mainly focused on protein-coding genes. While there are a few studies describing ncRNAs in several species, namely *C. albicans, C. parapsilosis,* and *C. glabrata*, they were mainly focused on short non-coding transcripts such as small nucleolar RNAs^62–64^. The repertoires of lncRNAs across *Candida* pathogens have never been thoroughly studied, which prevents us from fully understanding their potential roles in fungal virulence.

Here, we apply large-scale comparative transcriptomics and genomics to identify and analyze lncRNAs in five major *Candida* pathogens using a vast dataset of more than 4600 RNA-Seq and DNA-Seq samples. We characterize the main properties of the identified lncRNAs, assess their evolutionary relationships and potential functional roles. Finally, we investigate the expression of these transcripts throughout the course of epithelial infection, revealing transcripts potentially involved in pathogenicity.

## Results and discussion

### Inference and characterization of lncRNA catalogs in *Candida* spp

**Fig. 1:**
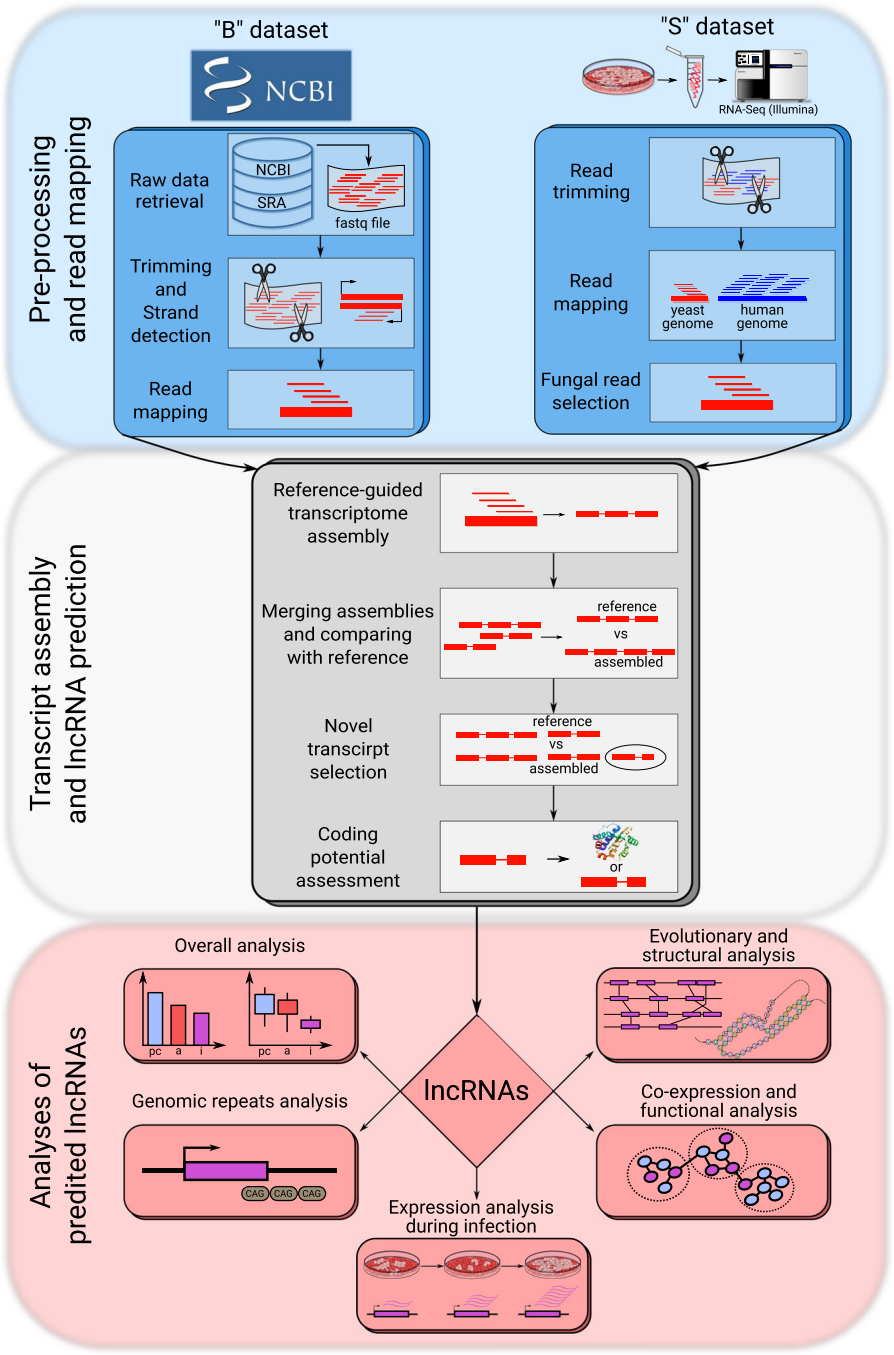
Schematic representation of the bioinformatics workflow for lncRNA prediction and analysis. See description in the text for details.

To infer and characterize lncRNA catalogs of the five major *Candida* pathogens, we used 2645 samples (see Supplementary Data 1 for the full list of samples with corresponding experimental and quality control information) comprising all publicly available RNA-Seq sequencing libraries for these species (Broad dataset, “B”) and RNA-Seq data from a large-scale in vitro host-pathogen interaction study of the four species *C. albicans, C. tropicalis, C. parapsilosis, and C. glabrata* with human vaginal epithelial cells (Specific dataset, “S”, see Materials and Methods and^65^ for details). We performed genome-guided transcriptome assemblies for each individual species and for each of B and S datasets independently. Assembled transcriptomes from B and S datasets were merged for each species to produce a final catalog of predicted lncRNAs (Fig. 1, see “Materials and Methods” for details).

Based on their genomic coordinates, we classified lncRNAs into intergenic (“i”), i.e., transcripts that do not overlap with protein-coding genes or other features, and antisense (“a”), i.e., lncRNAs overlapping coding genes or other features on the opposite DNA strand. Full lncRNA catalogs for all species are available in Supplementary Data 2 and are deposited in Candidamine—an integrative data warehouse for *Candida* yeasts available at https://candidamine.org/.

Prior to investigating the lncRNA repertoires in more detail, we assessed the impact of the chosen data analysis pipeline (i.e., genome-guided assembly by Stringtie^66^) on the results of transcriptome assembly, which is the main prerequisite for robust lncRNA identification. To this end, we compared the transcriptome assemblies with those produced by an alternative de novo transcriptome assembly approach as implemented in Trinity^67^. We observed that both assemblers largely produced consistent results in terms of reconstructing the reference annotated features, although the genome-guided method employed here rendered assemblies with higher specificity, sensitivity, and lower fragmentation rate (see the results of these analyses in Supplementary Note 1). Although the reference annotated features used in the benchmark do not include lncRNAs, we consider that it is reasonable to expect a better performance of Stringtie across transcript classes. Therefore, given the overall better performance of Stringtie in reconstructing reference transcripts, the results of the genome-guided transcriptome assembly approach were used in all downstream analyses.

**Fig. 2:**
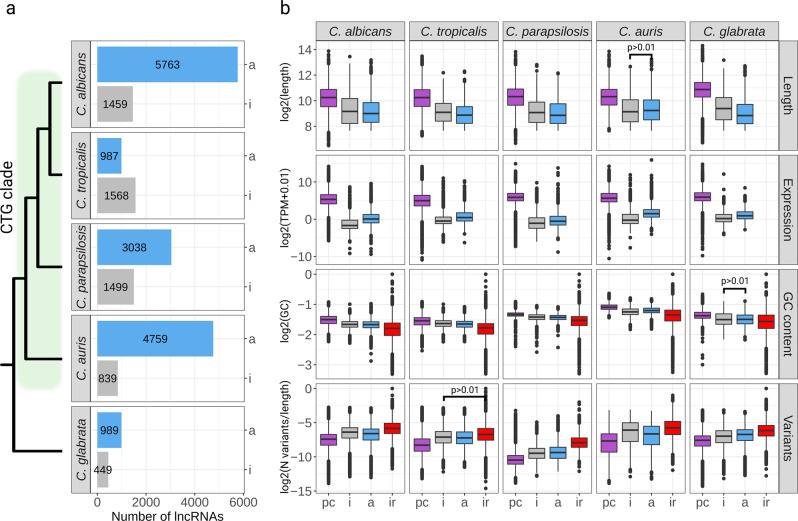
The lncRNA landscapes and their molecular properties in the analyzed *Candida* species. **a** Overall distribution of intergenic (“i’) and antisense (“a”) lncRNAs in the studied pathogens. Numbers on the bar plots indicate the corresponding number of lncRNAs. The schematic tree on the left side of the plot indicates phylogenetic relationships among the species. The green rectangle on the tree highlights the species of CTG clade; **b** Comparisons of transcript lengths, expression levels, GC content and sequence variation between protein-coding genes (“pc”), lncRNAs (“i” and “a”), and intergenic regions (“ir”) (where applicable) across *Candida* species. Differences between pairs of box plots within each species are statistically significant (Wilcoxon rank sum test *p* value < 0.01), unless indicated otherwise.

The largest number of lncRNAs (Fig. 2a) was detected in *C. albicans*, with 5763 antisense and 1459 intergenic transcripts, followed by *C. auris* (4759 and 839, respectively), *C. parapsilosis* (3038 and 1499), *C. tropicalis* (987 and 1568), and *C. glabrata* (989 and 449). For species of the CTG clade (i.e., all except *C. glabrata*), differences in the number of lncRNAs are mainly driven by the large number of antisense transcripts found in *C. albicans* (5763) and *C. auris* (4759) as compared to *C. parapsilosis* (3038) and *C. tropicalis* (987).

Of note, such drastic differences are not observed for *C. albicans* when analyzing only the samples of the host-pathogen interaction study (S dataset, 1088, 627, 968 antisense lncRNAs in *C. albicans*, *C. tropicalis,* and *C. parapsilosis*, respectively). Both biological differences and differences in the diversity of analyzed samples (Supplementary Data 1 and Figure S1) can potentially influence the number of lncRNAs detected in our study. An additional parameter that can have a strong effect on the amount of identified lncRNAs is the number of analyzed samples used for our final transcriptome assemblies, which was largest for *C. albicans* (*n* = 699), followed by *C. parapsilosis* (*n* = 86), *C. auris* (*n* = 61), *C. tropicalis* (*n* = 53), and *C. glabrata* (*n* = 51). To assess the impact of the difference in the number of analyzed samples, we repeated the lncRNA predictions using sample subsets and produced saturation plots showing the dependency of the number of analyzed samples and the number of predicted lncRNAs (Supplementary Fig. S2). This analysis showed that the number of antisense lncRNAs in *C. albicans* reaches a plateau for subsets of ~200 samples or larger. This suggests that few novel antisense lncRNAs in *C. albicans* may remain to be discovered. For the other species, considering they have at most 86 samples (for *C. parapsilosis*), it is likely that their antisense lncRNA catalogs are not complete, and might be expanded with new datasets. However, additional biological and technical factors that might influence this result include pervasive transcription, transcriptional noise, and varying efficiency of strand-specific library preparation protocols. On the other hand, extensive antisense transcription has been previously observed in *C. albicans* and *S. cerevisiae*, although to a lesser extent than observed in our study^62,68^. Additionally, it must be noted that our analyses are based on the use of reference genomes and therefore it is unclear how genomic variability across strains of the same species may impact lncRNA predictions. However, this problem is inherent to any reference-based inference, and our approach can be extended to multiple (i.e., clade-specific) reference genomes

While the number of antisense lncRNAs varied significantly across the four species of the CTG clade, the number of intergenic lncRNA was similar across the three more closely related representatives of this clade (1457–1581), with *C. auris* having a somewhat lower number (842). In contrast, the distantly related *C. glabrata* has a much lower number of intergenic lncRNAs compared to all other species (444). A lower number of intergenic lncRNAs in *C. glabrata* is also apparent when restricting the analysis to the S dataset (267, compared to 1223, 943 and 794 for *C. albicans*, *C. tropicalis,* and *C. parapsilosis*, respectively). Thus, the considerably lower number of intergenic lncRNAs in *C. glabrata* may reflect a true biological difference, in accordance with the large phylogenetic distance between *C. glabrata* and the other considered species^52^. Finally, saturation plots for intergenic lncRNA reached a plateau with only 30–40 samples, suggesting that the obtained catalogs are comprehensive. Of note, a decay in the total number of intergenic lncRNAs is observed in *C. albicans* as more datasets are analyzed, likely resulting from the fusion of previously fragmented transcripts.

Further, we assessed the distribution of lncRNAs along chromosomes (Supplementary Figs. S3 and S4). For *C. albicans, C. parapsilosis,* and *C. auris*, antisense transcripts were relatively evenly distributed across the chromosomes, as compared to more variable distribution in *C. tropicalis* and *C. glabrata* (Supplementary Fig. S3). Intergenic transcripts were unevenly distributed in all species, with some apparent hotspot regions. For example, in chromosomes 1A and 2A of *C. albicans*, these hotspots are observed near centromeres. In contrast, *C. glabrata* shows high accumulation of intergenic lncRNAs at the terminal (telomeric) sites of some chromosomes, such as chromosomes D, E, F, H, and J. This pattern of lncRNA hotspots (both intergenic and antisense) in *C. glabrata*, but not in other species was also clearly observed when calculating the number of lncRNAs relative to the distance to the closest telomere across all chromosomes (Supplementary Fig. S4).

We then assessed several major features of lncRNAs and compared them to those of protein-coding genes and, where available, previously annotated lncRNAs. Consistent with studies in other organisms^47,48^, we found that, compared to protein-coding genes, both lncRNA types tend to be shorter, have lower GC content (albeit higher than in intergenic regions), and lower levels of expression (Fig. 2b, see Supplementary Data 3 for the mean values of all parameters across species and comparisons). We further used population-scale variant calling data of each species derived from 1976 publicly available genome sequencing samples (see “Materials and Methods” for details) to analyze the variability of lncRNAs and compare it to protein-coding genes and intergenic regions.

The analysis of genetic variants (Fig. 2b) showed that lncRNAs accumulate more variants than protein-coding genes but significantly fewer than intergenic regions (with an exception of *C. auris* where intergenic lncRNAs had higher variability than intergenic regions, see Supplementary Data 3), indicating that lncRNAs are evolutionarily constrained.

We also performed a similar set of analyses for 17 previously predicted lncRNAs in *C. glabrata*^64^, and observed that these transcripts were generally more similar to protein-coding genes than to lncRNAs found in our study (Supplementary Fig. S5). This might be explained either by the small sample size of previously annotated lncRNAs, or their possible misidentification as noncoding transcripts.

It has been previously shown that lncRNAs in a wide range of taxa can encompass transposable elements (TEs) and genomic repeats, which can influence their origin, architecture, evolutionary trajectories, and regulation^69–71^. We, therefore, analyzed whether and to which extent lncRNAs in *Candida* pathogens harbor these elements and compared it to protein-coding genes (Supplementary Fig. S6). This analysis (see “Materials and Methods” for details) showed that many lncRNAs, both intergenic and antisense ones, harbor repeat regions in all studied species, although to a different extent. The highest values were observed in *C. tropicalis* (50.5% of intergenic and 50.9% of antisense lncRNAs) and *C. albicans* (48.5% of intergenic and 50.8% antisense lncRNAs) and were decreasing following the phylogenetic relationships of the species, reaching in *C. glabrata* to 20.7% of both intergenic and antisense lncRNAs. By far the most prevalent type of repeats found within lncRNAs are simple repeats, followed by low complexity repeats, while the prevalence of LINE, SINE, and LTR repeats was generally low. Importantly, for all species except *C. glabrata*, we also observed a high proportion of protein-coding genes overlapping repeats, which however was lower than in the case of lncRNAs. For example, for *C. albicans* 40.1% of protein-coding genes overlapped repeats. Of note, this pattern of having more repeat regions in lncRNAs than in protein-coding genes has been reported in a wide range of taxa^69,72,73^.

### Classification of lncRNAs into conserved families

We next explored the evolutionary relationships of lncRNAs across the studied species. Considering that lncRNAs generally show low levels of sequence conservation and may adopt conserved secondary structures, we used three alternative approaches to establish their potential relatedness: sequence similarity, structural similarity, and synteny. This is specifically relevant here, as the studied *Candida* species are phylogenetically very diverse. As antisense lncRNAs partially overlap protein-coding genes, we excluded them from this analysis. We first identified one-to-one best reciprocal BLAST hits between each pair of species (see Materials and Methods for details) and used a previously developed clustering methodology^48^ to unify all pairwise species comparisons and define lncRNA families across species. As expected, this analysis identified a very small number of conserved lncRNAs families—merely one between *C. tropicalis* and *C. parapsilosis* (see Supplementary Fig. S7) containing one lncRNA from each species (MSTRG.4971.1 and MSTRG.364.1, respectively). To validate this result with a different clustering approach, we ran OrthoMCL analysis using Synima software^74,75^, which rendered the same lncRNA family as obtained by BLAST reciprocal hits. These results highlight the overall low sequence conservation of lncRNAs, and the high levels of species divergence.

We then analyzed structural similarities of lncRNAs using the Beagle software^76^ which performs pairwise alignments of secondary structures (see “Materials and Methods” for details). In this approach the majority of lncRNAs were classified into structural families (~98.2%, Supplementary Fig. S7) and most families (84.13%) were shared by all five species, which is in stark contrast with BLAST results. It was unclear, however, whether this similarity is the result of higher evolutionary constraints in the structure^17,77^, or whether it can be attributed to a low specificity of the structural comparison approach. To test this, we repeated the above mentioned analysis 50 times using randomly reshuffled sequences of lncRNAs, and observed similarly large fraction of lncRNAs classified into families (mean = 97.11%, *p* = 0.86 compared to the real rate), indicating that the alignment of secondary structures with Beagle has low specificity and does not allow drawing evolutionary inferences.

**Fig. 3:**
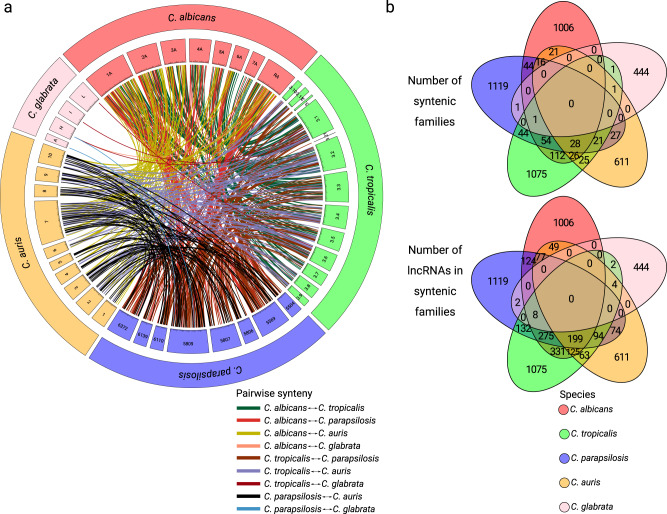
Assessment of syntenic relationships of intergenic lncRNAs across *Candida* species. **a** An aggregated circos plot providing a general overview of all pairwise syntenic relationships between lncRNAs of the studied species. Exact locations of all syntenic lncRNAs are available at Supplementary Data 4; **b** Venn diagrams representing the number of classified syntenic lncRNAs families (at the top) and the number of corresponding lncRNAs within families (at the bottom) across the species.

We finally searched for syntenic lncRNAs using a methodology developed and validated in^48^ (see “Materials and Methods” for details). This analysis revealed a significantly higher number of evolutionary related lncRNAs (*n* = 881, 26.8%) than the BLAST-based approach, but considerably lower than in the case of secondary structure analysis (Fig. 3, see Supplementary Data 4 for pairwise synteny information across species). We repeated this analysis with varying levels of stringency of synteny classification parameters and consistently obtained similar results (Supplementary Data 5 and 6). As expected from their closer evolutionary relationships, the majority of the syntenic relationships were observed between species of the CTG clade, while the level of synteny between *C. glabrata* and other species was lower.

We further inspected syntenic families for the presence of conserved sequence motifs shared by transcripts within a family, which could additionally support functional or evolutionary relatedness. Motif discovery analysis using MEME^78^ detected 91 families (or 21.5%) with shared conserved motifs. This fraction of lncRNA families with shared motifs was significantly higher than random expectation (*p* < 0.05) as assessed in a set of randomly generated families (see “Materials and Methods”) which on average achieved a 0.74% rate of motif discovery. A similar analysis using an independent graph-based approach implemented in lncLOOM^79^ showed that 85 families (20%) had shared sequence motifs. Additionally, the same analysis using random families showed a significantly lower rate (8.5%, *p* < 0.05). We then compared the results of the two approaches and found 33 lncRNA families which were identified to have conserved sequence motifs by both tools. Further analysis indicated that despite the significant difference between computational methodologies employed by MEME and lncLOOM, there were 5 lncRNA families where motifs found by the two software were overlapping.

Overall, our results suggest that despite large evolutionary distances between *Candida* species and the overall poor sequence conservation, these pathogens share syntenic lncRNAs which possess short patches of conserved sequence motifs. Such short patches of conserved sequences in lncRNAs have been broadly reported (see^15,80^ for recent reviews), inspiring the “RNA modular code” hypothesis^81^, which posits that these elements can form discrete functional secondary and tertiary structures mediating interactions with other molecules. Testing this requires a combination of complex experimental and bioinformatics analyses. In this context, our findings of syntenic lncRNA families with conserved sequence motifs in major *Candida* pathogens open novel opportunities for disentangling those relationships in a targeted manner for future research.

### Co-expression analysis

The potential functions of the newly identified lncRNAs are unknown, but their patterns of co-expression with protein-coding genes of known function can hint on possible roles^82,83^. Considering this, we carried out a gene co-expression network analysis for intergenic lncRNAs and protein-coding genes using the WGCNA approach (see “Materials and Methods”). After inspecting principal component analysis plots (Supplementary Fig. S1) to remove outliers and filtering out lowly expressed genes (TPM < 0.1 in more than 80% of samples), we obtained sufficient power values (*β* = 12–16) to generate scale-free co-expression networks for each of the species (Supplementary Fig. S8).

For all species, a co-expression network analysis identified multiple highly interconnected gene modules (*n* = 9–20, Supplementary Fig. S9). Interestingly, lncRNAs were present in the majority of modules, with the only exception of *C. albicans*, indicating that lncRNAs are common members of co-expressed gene clusters. Despite being widely distributed across networks, lncRNAs have significantly lower co-expression connectivities than protein-coding genes (Wilcoxon rank sum test *p* value < 0.05). Despite this generality, lncRNAs MSTRG.7139.1 of *C. tropicalis* and MSTRG.4801.1 of *C. glabrata* were the most highly connected nodes (i.e., the hubs) in their corresponding modules (Supplementary Fig. S7).

To gain functional insights on the lncRNAs involved in modules, we performed enrichment analyses of GO terms, KEGG pathways, and PFAM domains of the protein coding genes in all modules (see Supplementary Data 7 for all identified enrichments of each module and species). We observed a wide variety of enrichments across all the modules, including terms related to fungal pathogenicity, such as “adhesion of symbiont to host” and “pathogenesis” for *C. albicans* (module “darkred”) and “filamentous growth” for C. *tropicalis* (module “coral1”).

### Identification of lncRNAs specifically regulated during epithelial infection

**Fig. 4:**
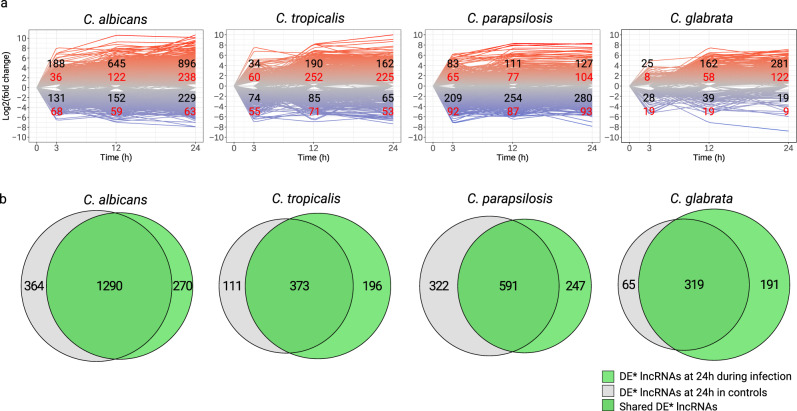
lncRNAs during fungal infection of epithelial cells. **a** lncRNAs expression dynamics plots based on log2 fold changes compared to time point 0. Each line (up-regulated—red, down-regulated—blue) corresponds to the fold change of expression levels of a lncRNA. Numbers on the plots indicate the number of differentially expressed lncRNAs (|log2 fold change | >1.5, padj < 0.01, in red—intergenic lncRNAs, in black—antisense lncRNAs); **b** Venn diagrams of differentially expressed* (DE*) lncRNAs during infection (in green) and in control samples (in blue). *To identify infection-specific genes with a higher stringency, we applied filters of |log2 fold change | >0 and padj < 0.01 to the DE lncRNA sets in both conditions in order to maximize the overlap between them, hence ensuring that lncRNAs in control samples even with small fold-changes are discarded. The numbers of “infection-specific” lncRNAs are indicated in green-only portions of Venn diagrams.

To investigate in more detail the possible implication of *Candida* lncRNAs in virulence, we resorted to the S dataset, which comprises host-pathogen expression data through the time-course of epithelial infection of four of the studied pathogens—*C. albicans, C. tropicalis, C. parapsilosis,* and *C. glabrata*. To this end we performed a differential expression analysis to identify lncRNAs (both intergenic and antisense) which are differentially expressed throughout the infection (see Supplementary Data 8–11 for data for *C. albicans*, *C. tropicalis*, *C. parapsilosis,* and *C. glabrata*, respectively). As shown in Fig. 4a, there are numerous lncRNAs that significantly change their expression upon interaction with epithelial cells from the initial time point of infection, with the number of differentially expressed lncRNAs increasing as the infection progresses.

The 24 h control samples present in the S dataset allowed us to identify lncRNAs which are differentially expressed in the absence of human epithelial cells, accounting for the effect of time and changes in the growth medium. We observed that the process of infection and normal growth in culture medium deregulate largely overlapping sets of lncRNAs (Fig. 4b). This phenomenon has been already observed for protein-coding genes of these species^65,84^, indicating that most non-coding and coding genes related to pathogenesis of these species are also related to the standard growth metabolism. We nevertheless identified a substantial number of infection-specific lncRNAs, which are differentially expressed exclusively due to the infection process (*n* = 191–270, depending on the *Candida* species).

**Fig. 5:**
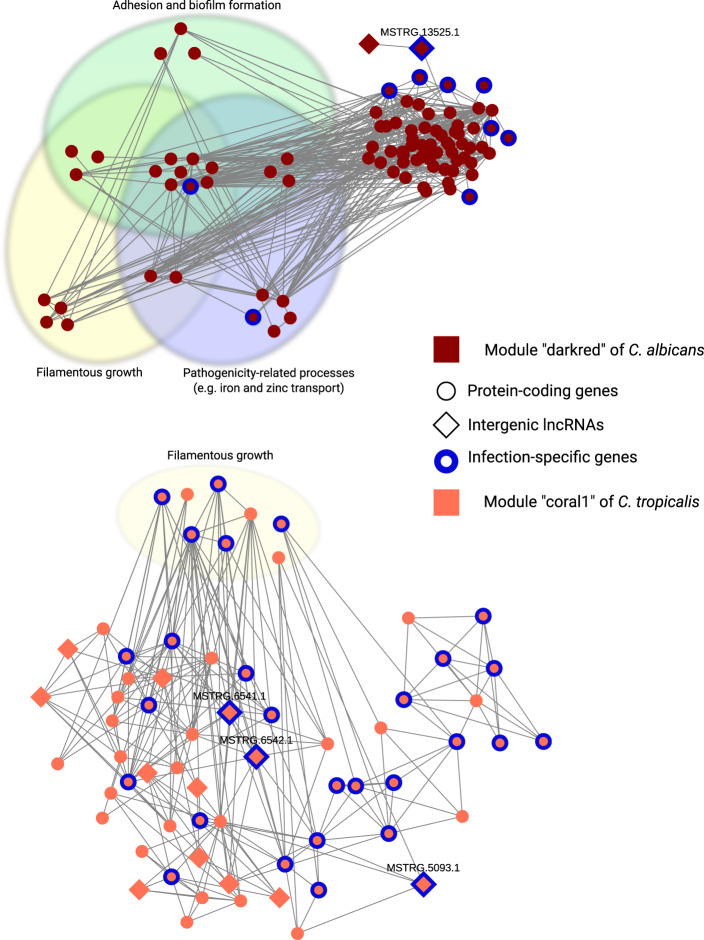
Network modules with infection-specific lncRNAs and GO term enrichments related to fungal virulence. Module “darkred” of *C. albicans* (at the top), showing nodes with WGCNA weight > 0.01 and degree ≥ 1, for better visibility. Module “coral1” of *C. tropicalis* (at the bottom), showing nodes with WGCNA weight >0.1 and degree >3. Circular nodes represent protein coding genes; diamond-shape nodes represent lncRNAs; Nodes highlighted with colored ovals correspond to genes with significant GO term enrichments related to fungal pathogenicity. Edges represent significant correlations as estimated by WGCNA; colors of nodes correspond to the module names assigned by WGCNA software (“darkred” and “coral1” for *C. albicans* and *C. tropicalis*, respectively); nodes with blue borders represent infection-specific genes (infection specific protein-coding genes are obtained from^65^); Identifiers of infection-specific lncRNAs are mentioned at the top of corresponding nodes. Nodes which are not assigned to virulence-related GO terms are positioned according to Prefuse Force directed layout.

Further analysis of the “infection-specific” lncRNAs showed no significant differences between their network connectivities compared with other lncRNAs. Additionally, we observed that a large portion of “infection-specific” intergenic lncRNAs (~10–50%) are involved in modules. Of note, modules containing infection-specific lncRNAs had various enrichments (see Supplementary Data 7), some of which were directly related to pathogenicity, such as “pathogenesis” and “adhesion of symbiont to host” for *C. albicans* (module “darkred”) and “filamentous growth” for *C. albicans* (module “darkred”) and *C. tropicalis* (module “coral1”) (Fig. 5). To ensure that the co-expression signals of “infection-specific” lncRNAs in these modules are not spurious, we investigated the expression levels of the “infection-specific” lncRNAs with the highest WGCNA weight and corresponding co-expressed coding genes across the analyzed samples, which reinforced their co-expression patterns (Supplementary Fig. S10). Interestingly, this analysis also showed that, in *C. tropicalis* “coral1” module, the protein coding gene which is co-expressed with the two infection-specific lncRNAs with highest weights (i.e., MSTRG.6541.1 and MSTRG.6542.1) is an infection-specific protein coding gene CTRG_00938 (as identified by Pekmezovic et al.^65^). This gene has an unknown function in *C. tropicalis*, but has an ortholog in *C. albicans* (CR_01500W) which is implicated in filamentous growth (according to Candida Genome Database^85^).

We then assessed syntenic relationships of “infection-specific” lncRNAs across species (Supplementary Fig. S11), which showed the presence of a few syntenic families sharing infection-specific lncRNAs between closely related species of CTG clade. Namely, there were three families between *C. albicans*, and *C. tropicalis*, one family *C. albicans* and *C. parapsilosis* and two families between *C. tropicalis* and *C. parapsilosis*. Overall, this analysis suggests that infection-specific genes tend to be specific for each *Candida* pathogen, in agreement with previous results for protein coding genes^65^.

### Concluding remarks

Despite intensive research on lncRNAs during the recent decade^86^, these enigmatic transcripts have never been systematically investigated in human fungal pathogens from the *Candida* clade. Here, we mined and analyzed thousands RNA-Seq samples available for the studied yeasts, and reported comprehensive lncRNA catalogs of the five major *Candida* pathogens. As in other eukaryotic species, lncRNAs are abundant in these *Candida* yeasts. We classified the identified lncRNAs into intergenic and antisense transcripts, and show that antisense transcription of lncRNAs is widespread in *Candida*. These catalogs constitute a valuable resource to guide further research into the role of lncRNAs in these organisms. Incidentally, one of our predicted lncRNAs in *C. auris*, namely MSTRG.10503.1, was experimentally identified and characterized in an independent study^87^ while our manuscript was in revision. This lncRNA, named DINOR in that study, was shown to act as a virulence factor and a regulator of various stress responses in *C. auris*. This independent confirmation of one of our predicted lncRNAs attest for the validity of our approach for identifying biologically relevant lncRNAs.

We found that *Candida* lncRNAs exhibit similar general properties as lncRNAs in other species, i.e., they are shorter, and have lower GC content, expression levels and evolutionary constraints when compared to protein-coding genes. From the evolutionary standpoint, lncRNAs in *Candida* show poor primary sequence conservation, but importantly they accumulate less sequence variation than intergenic regions, which hints to the existence of selective pressures acting on these transcripts. Moreover, lncRNAs showed detectable levels of synteny between CTG clade members reflecting their phylogenetic relationships. In fact, these properties of lncRNAs seem to be universal across plants, animals and fungi^46–48,88^, indicating common evolutionary constraints.

Co-expression network analysis revealed that lncRNAs of *Candida* are ubiquitously co-expressed with protein coding genes. Considering that highly co-expressed features are likely to be functionally related^89^, we show that lncRNAs can potentially have numerous functional implications in *Candida*, including virulence.

We further specifically investigated the participation of the predicted lncRNAs in virulence processes by assessing their expression dynamics during the course of human epithelial cell infection. For each species, we identified a large number of infection-specific lncRNAs which were differentially expressed exclusively due to interaction with the human host, and were co-expressed with genes related to fungal virulence. These transcripts can be considered as direct targets for further experimental analysis. Altogether, the lncRNAs catalogs inferred here serve as a valuable resource and open novel avenues for further research in human yeast pathogens.

## Methods

### Datasets

We used two datasets to define the lncRNA landscapes of the studied yeasts: one comprising all RNA-Seq data publicly available at the Sequence Read Archive (SRA) database^90^, and another one comprising a single large-scale RNA-Seq experiment including the four species *C. albicans, C. tropicalis, C. parapsilosis and C. glabrata*^65^. Hereafter, we refer to these datasets as B (Broad) and S (Specific), respectively.

The B dataset was retrieved from SRA database (last accessed on 19th of July 2019^90^,) using sratoolkit v. 2.9.6-1 with prefetch and fastq-dump functions. In total, sequencing data from 2561 libraries were downloaded (see Supplementary Data 1), of which 2177 for *C. albicans*, 129 for *C. parapsilosis*, 123 for *C. glabrata*, 86 for *C. auris*, and 46 for *C. tropicalis*. FastQC v. 0.11.6 (https://www.bioinformatics.babraham.ac.uk/projects/fastqc/) and Multiqc v. 1.0^91^ were used to perform quality control of raw sequencing data. For *C. albicans*, we discarded 64 libraries with read length shorter than 49 bp. The remaining samples were pre-processed to obtain high-quality data. First, we trimmed all samples using Trimmomatic v. 0.36^92^ with the following parameters: <ADAPTERS.fa>:2:30:10 LEADING:3 TRAILING:3 SLIDINGWINDOW:4:15 MINLEN:49. Then, we assessed the strand-specificity of the libraries by first running RSEM prepare-reference v. 1.3 to extract the transcriptomes of the analyzed species and then running salmon v. 0.8.1, which identifies the strandedness of the data^93^. We then discarded non-strand-specific libraries. This step ensures that expression of lncRNAs is not confounded by reads corresponding to other features (protein coding genes, tRNAs, rRNAs, etc.) located on the opposite DNA strand. With the remaining data, i.e., 666 libraries for *C. albicans*, 35 for *C. tropicalis*, 71 for *C. parapsilosis*, 61 for *C. auris* and 37 for *C. glabrata*, we performed read mapping to the corresponding reference genomes using TopHat2 v. 2.1.1 with *--b2-very-sensitive* option^94^. Reference genomes and annotations for *C. albicans* SC5314 (assembly 22), *C. glabrata* CBS138, *C. parapsilosis* CDC317, *C. auris* B8441 and *C. tropicalis* MYA-3404 were obtained from *Candida* Genome Database (CGD, last accessed on 17 of August 2017^85^). Considering that the genome sequence of *C. albicans* is phased, in our analysis we used only haplotype A for read mapping to avoid a substantial amount of multi-mapped reads.

The S dataset corresponds to a previous RNA-Seq study of the interaction between human vaginal epithelial cell line A451 and the four *Candida* species^65^ *C. albicans, C. tropicalis, C. parapsilosis* and *C. glabrata*. The dataset comprises samples taken at 1.5, 3, 12, and 24 h post-infection, and includes controls for the effect of the culture medium on the fungal transcriptional activity. In total, the S dataset comprised 84 samples—37 libraries for *C. albicans*, 18 for *C. tropicalis*, 15 for *C. parapsilosis,* and 14 for *C. glabrata*, representing strand-specific sequencing libraries with 2 × 50 and 2 × 75 bp read length. The samples which had traces of adapter sequences and/or poor quality bases were trimmed with Trimmomatic using <ADAPTERS.fa>:2:30:10 LEADING:1 TRAILING:1 SLIDINGWINDOW:4:1 MINLEN:<50,75> command. Subsequently, since most of the S dataset libraries comprised dual RNA-Seq data (i.e., mixed human and fungal RNA), we mapped the data to the concatenated reference genome of each fungus and human. Human reference genome GRCh38 and annotations were obtained from Ensembl database release 89 (last accessed on 8 of August 2017^95^). Then, we generated fungus-only bam files by subsetting mapped fungal reads from the pooled bam files using samtools v. 1.3.1^96^.

Detailed information about all RNA-Seq samples used in this study is available in Supplementary Data 1.

### Computational prediction of lncRNAs

For each sample we performed genome-guided transcriptome assembly using Stringtie v. 1.3.3b^66^. We then compared the result of Stringite-based genome-guided transcriptome assembly with de novo transcriptome assemblies reconstructed by Trinity v. 2.8.5 software^67^, using the available reference annotations from CGD as gold standard. This comparison showed overall consistent results between the two softwares, and revealed that genome-guided Stringtie assembly resulted in higher specificity, sensitivity and lower fragmentation rate of the overall transcriptome reconstruction (see the Supplementary Note 1 for the detailed description of the performed comparisons). For this reason, all downstream analyses for lncRNA identification were performed using Stringtie results.

Further, for each species we merged all assembly gtf files produced by Stringtie from all samples using Stringtie merge with *-g 50* option which resulted in a unified transcriptome. These transcriptome annotations were compared with the original genome annotations of each species using gffcompare v. 0.11.2 to identify novel transcripts.

For *C. albicans*, our initial mapping and assembly strategy resulted in transcripts with artifactually long introns spanning several hundred thousand bases. To avoid this, we repeated the analysis for all species setting the TopHat2 option --max-intron-length to 1000, which corresponds to approximate maximum intron length in fungal genomes^97^. Additionally, for *C. albicans* we removed four assemblies from the project PRJNA292429 (assembled from SRR2153488-SRR2153491 [https://www.ncbi.nlm.nih.gov/sra?linkname=bioproject_sra_all&from_uid=292429] accession numbers), which were producing long intergenic transcripts compared to the other datasets thereby resulting in the bridging of novel intergenic transcripts with coding genes during the transcriptome merging step.

Next, we selected novel intergenic (“i”) and antisense (“a”) transcripts (corresponding to “u” and “x” class-codes of gffcompare output, respectively) longer than 200 bp. When several isoforms were present, we kept only the longest one using CGAT gtf2gtf v.0.3.2 software^98^. Further, we assessed the coding potential of the predicted transcripts using the CPC v. 0.9^99^ and Feelnc v. 0.1.1^100^ software. CPC was run against the UniProt database (https://www.uniprot.org/downloads, last accessed on 9 July 2019), and the output transcripts assigned with “noncoding” label were retained. For Feelnc, we used the sequences of protein coding genes with *shuffle* mode as training datasets for its Random Forest machine learning algorithm. Additionally, for *C. albicans* and *C. tropicalis* we removed transcripts (*n* = 438 and *n* = 4, respectively) containing ambiguous nucleotides because they produced errors in the software runs. The coding potential cut-offs were defined using a tenfold cross-validation, as implemented in Feelnc. Transcripts identified as non-coding by both software tools were considered as lncRNAs. In addition, the transcripts discarded from Feelnc runs due to the presence of ambiguous nucleotides but identified as non-coding by CPC were included in our final lncRNA datasets.

After obtaining the sequences of lncRNAs across species, we ran BLASTn v.2.9 (with -max_target_seqs 5, -max_hsps 5 and -evalue 1e-3) of intergenic lncRNA catalogs of each species against genomes of other species to test if any intergenic lncRNAs matched with annotated features or lncRNAs in other genomes. The analysis was restricted to intergenic transcripts since the sequence similarity between the known features might influence the results obtained for the antisense transcripts. The BLAST results (Supplementary Data 12 and 13) were then converted to bed format and compared with reference features and lncRNAs of every other species using bedtools intersect, requiring at least 50% match of the query lncRNAs. After manual inspection of the results, we identified 13 lncRNAs in *C. tropicalis* and 3 lncRNAs in *C. auris* that were matching to rRNA/tRNA/ncRNA of other species, and hence discarded these 16 transcripts from further analysis (see Supplementary Data 14).

Analysis of distribution of lncRNAs across chromosomes was done using chromPlot v. 1.14.0^101^

### Overall expression levels of lncRNAs

To assess the overall expression levels of lncRNAs and compare them to those of protein-coding genes, we calculated the read counts of both transcript categories in all analyzed samples using Featurecounts v. 1.6.4^102^. The count data were normalized by transcript length and library size, resulting in transcripts per million (TPM) values.

### Analysis of sequence variation

To assess the variability of lncRNA sequences and compare it with that of protein-coding genes and intergenic regions, we utilized variant calling data of the studied species available in the Candidamine database at https://candidamine.org/, which comprises variants obtained from all 1976 publicly available DNA sequencing data of these yeasts. In particular, we used variants from 652 samples of *C. albicans*, 420 samples of *C. glabrata* and 51 samples of *C. parapsilosis*, 764 samples of *C. auris,* and 89 samples of *C. tropicalis*. Using bedtools v.2.29.2^103^ with *intersect* function we calculated the number of variants located in both types of lncRNAs, protein coding genes, and intergenic regions across the studied species. Intergenic regions were retrieved using bedtools *complement* function applied to genome annotation files obtained after gffcompare.

### Analysis of genomic repeats and TE elements

Repeat calling was performed using perSVade pipeline v. 0.10 (https://github.com/Gabaldonlab/perSVade), which runs RepeatModeler v. 2.0.1^104^ and RepeatMasker v. 4.0.9 (Smit, AFA, Hubley, R & Green, P. RepeatMasker Open-4.0. 2013-2015, http://www.repeatmasker.org) (both with default parameters) on the query genomes, and reports a table of found repeat regions. For this study we analyzed the following types of repeats—simple repeats, low-complexity repeats, LTRs, LINE/SINE and repeats classified as unknown by Repeatmodeler/Repeatmasker. We then used bedtools intersect to calculate the proportion of each lncRNA type and protein coding genes overlapping different classes of found repeat regions, requiring at least 50% of a given repeat’s sequence to overlap lncRNA/protein-coding gene.

### lncRNAs gene family classification

To assess evolutionary relationships between predicted lncRNAs across species, we used several independent strategies, namely blast reciprocal hits, secondary structure similarity, and analysis of synteny. Considering that overlapping features of antisense lncRNAs can potentially influence the results, only intergenic transcripts were used for these analyses. To define best reciprocal hits between all possible pairs of species we used BLASTn v.2.9. To this end, we built a custom BLAST database for the set of lncRNAs of each species. Then, each set of lncRNAs was aligned against each database with BLASTn using cut-off of e-value < 1e-3, and -max_hsps 1 and -max_target_seqs 1, which selects only the best alignment between matched query-sequence pair. Best reciprocal hits were selected. We also repeated this analysis with parameters -max_hsps 10 and -max_target_seqs 10 and obtained the same results. Additionally, to test a different clustering approach, we have run OrthoMCL analysis^75^ using Synima pipeline (downloaded on August 1st, 2021^74^).

To assess the relatedness of the lncRNAs based on their secondary structures, we first used RNAfold v. 2.4.14 from the ViennaRNA package^105^ to obtain secondary structures for all studied intergenic lncRNAs. Then, using Beagle v. 0.2^76^ with the local alignment mode, the lncRNAs structures of each species were aligned against those of other species in a pairwise manner. Similarly to BLASTn best reciprocal hits approach, for each lncRNA we selected the hits with maximal zScore (at least z Score > 3) and *p* < 0.01. Reshuffling of lncRNA sequences for testing the specificity of secondary structure alignments was done using fasta-shuffle-letters from MEME suit v. 4.11.2^78^.

We classified intergenic lncRNAs into syntenic transcripts using a methodology developed and validated in^48^. Briefly, we first obtained the information of 1-to-1 orthologs between protein-coding genes in the four *Candida* species from CGD and CGOB^106^. Then, we defined pairwise syntenic relationships between the lncRNAs of the studied species using the *synteny_nematodesv4GH.py* script from^48^ with modifications directed to the analysis of five instead of four species, and also to correctly match the species names and transcript identifiers in our study. The script was run with parameters *3 3 1*, i.e., considering three protein coding genes at each side of a given lncRNA, a minimum of three shared genes for each pairwise comparison between species, and a minimum of one shared gene at each side of a lncRNA. The analysis identified syntenic lncRNAs between species. We also performed the synteny classification with varying stringency of the above mentioned parameters (see Supplementary Data 5 and 6).

Finally, to cluster best reciprocal hits, results of secondary structure alignments and pairwise syntenic lncRNAs into lncRNA families across species, we used *classifyFamiliesv5_VennGH.py* script from^48^ which was modified for our study. The modified versions of both scripts from^48^ are available at our GitHub page https://github.com/Gabaldonlab/lncRNAs.

### Analysis of sequence motifs in syntenic families

We assessed whether the lncRNAs of identified syntenic families shared sequence motifs within a family by employing two independent approaches. First, we scanned the sequences of lncRNAs of syntenic families for motifs using MEME v. 4.11.2^78^. Motifs with e-value < 0.05 were considered as significant. We considered a syntenic family to have a shared motif if all the members of the family contained at least one shared motif. To assess how the identified number of syntenic families with shared motifs was different from random expectation, we simulated the same number and size of lncRNA families by randomly choosing lncRNAs and performed the shared motif discovery as described above. The simulated families comprised transcripts randomly sampled from the whole set of intergenic lncRNAs, preserving the number of lncRNAs per species observed in the real syntenic families. We repeated this simulation 100 times and compared the observed rate of shared motifs with the distribution in randomly formed lncRNAs families. Second, we used the recently developed tool lncLOOM v. 1.0^79^, which uses a graph-based approach to identify short conserved motifs in evolutionary related sets of sequences. For each lncRNA syntenic family we have run lncLOOM with -r 100 option, letting the software to randomly simulate the input sequences to calculate *p*- and *e*-values of motif discovery. As in the case of MEME, a syntenic family was considered to have a shared motif if all the members of the family had at least one motif with p- and e-values < 0.05. Additionally, we have run lncLOOM on 100 randomly generated families and compared the results with those of the real dataset.

### Co-expression analysis

We assessed the patterns of gene co-expression across all intergenic lncRNA transcripts using the weighted correlation network analysis approach implemented in WGCNA v. 1.69^107^. As in case of gene family classification, antisense transcripts were discarded from this analysis. We used log2(TPM + 1) as expression values. For each species, we first selected the *β* power values using the “picksoftThreshold” function implying an unsigned network. The minimum *β* value reaching 80% of scale-free network topology, specifically *β* = 12 for *C. albicans*, *β* = 16 for *C. tropicalis*, *β* = 14 for *C. parapsilosis*, *β* = 12 for *C. auris,* and *β* = 12 for *C. glabrata*, was used for downstream analysis. To reach the optimal values of *β*, we removed samples comprising outliers as identified by inspection of principal component analysis (PCA) plots based on expression values (Supplementary Fig. S1). Namely, we removed samples of the following accession numbers: SRP099169 and SRP083839 for *C. tropicalis*, SRP151798 and SRP041812 for *C. parapsilosis* and SRP065276 for *C. glabrata*. Additionally, we removed all genes that had TPM values < 0.1 in more than 80% of the remaining samples.

We inferred modules in the WGCNA networks using 1-Topology Overlap Matrix values, and identified eigengenes (i.e., the first principal component of each module). Finally, we assessed network and module connectivities and identified hubs, as implemented in WGCNA with defaults parameters. For each identified module, we performed GO term, KEGG pathway and PFAM domain enrichment analysis of its protein-coding genes using clusterProfiler v. 3.14.3, selecting five enrichments with lowest adjusted p-values (at least *p* < 0.05). Adjustment of *p* values was done by the Benchamini-Hochberg procedure. For GO term enrichment analysis, we used “Biological Process” category. GO term association tables and protein domain predictions were obtained from CGD. All custom calculations were performed in R v. 3.6.1 using various packages. Network visualizations was done with Cytoscape v. 3.7.2.

### LncRNA expression during epithelial cell infection

We assessed the expression of all predicted lncRNAs during the infection model of vaginal epithelial cells interacting with four *Candida* species *C. albicans, C. tropicalis, C. parapsilosis and C. glabrata*^65^. For this, we first calculated the mapped read counts for lncRNAs and known features using Featurecounts v. 1.6.4^102^. For each species we performed differential expression analysis using DESeq2 v. 1.26.0 Bioconductor package^108^, by comparing each time point of infection with 0 h time point control samples using the Wald test. Differential expression calls were performed with the count data of both known features and lncRNAs. LncRNAs with |log2 fold change (L2FC) | > 1.5, and padj (adjusted *p* value) < 0.01 were considered as differentially expressed (unless specified otherwise). Correction of possible batch effects was done using the RUV v. 1.20.0 Bioconductor package.

### Reporting summary

Further information on research design is available in the Nature Research Reporting Summary linked to this article.

## Supplementary information

Supplementary Information

Description of Additional Supplementary Files

Supplementary Data 1

Supplementary Data 2

Supplementary Data 3

Supplementary Data 4

Supplementary Data 5

Supplementary Data 6

Supplementary Data 7

Supplementary Data 8

Supplementary Data 9

Supplementary Data 10

Supplementary Data 11

Supplementary Data 12

Supplementary Data 13

Supplementary Data 14

Reporting Summary

## Data Availability

All publicly available RNA-Seq datasets were retrieved from NCBI SRA database (see Supplementary Data 1 for details of all samples used in this study). Reference genomes, genome annotations, orthology information, GO and PFAM domain tables were obtained from Candida Genome Database. Variant calling data were obtained from Candidamine database. See “Methods” section for more details. All datasets generated in this study are available at our GitHub page https://github.com/Gabaldonlab/lncRNAs.

## Code availability

The whole data analysis pipeline, i.e., all scripts, datasets, annotations, software versions and auxiliary files to reproduce the results of the study, plots and supplementary materials, are available at our GitHub page https://github.com/Gabaldonlab/lncRNAs. Any updates to the codes and supplementary materials will be available in our GitHub page.
