# Supplementary material for: The long non-coding RNA landscape of Candida yeast pathogens
Source: Nat Commun. 2021 Dec 16;12:7317. doi: 10.1038/s41467-021-27635-4 (PMC8677757; doi:10.1038/s41467-021-27635-4)
Supplement: Supplementary file 1 — Supplementary Information [file 41467_2021_27635_MOESM1_ESM.pdf]

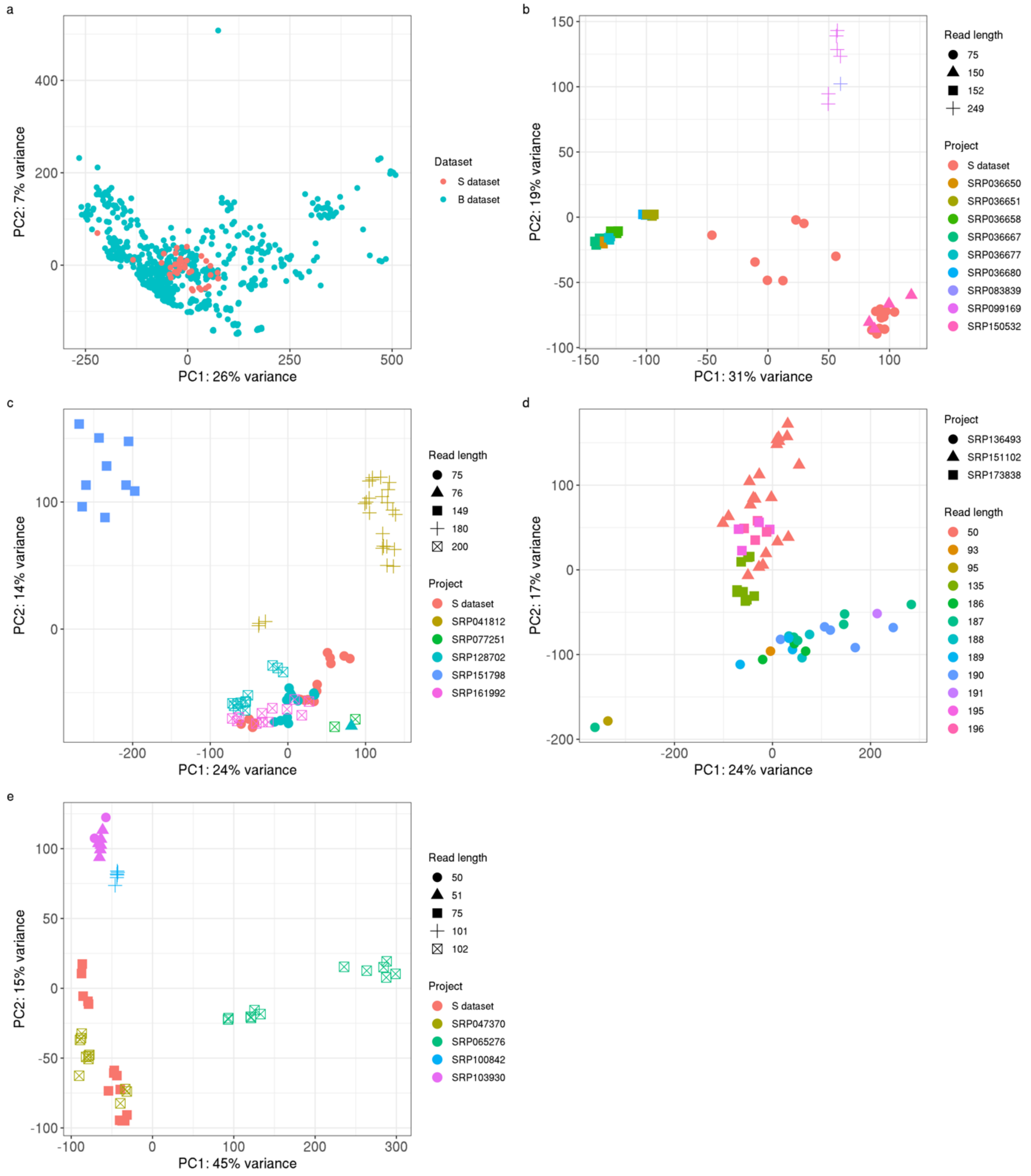

Figure S1: Principal Component Analysis (PCA) plots of all analyzed samples across species. Plots for **(a)** *C. albicans*; **(b)** *C. tropicalis*; **(c)** *C. parapsilosis*, **(d)** *C. auris* and **(e)** *C. glabrata*. Plots were generated using log2-transformed TPM expression values. Color codes correspond to SRA project accession numbers (or to sequencing read length for *C. auris*) and S dataset used in this study. Due to a large number of SRA projects and read lengths and for better visibility, the plot for *C. albicans* indicates only S and B datasets.

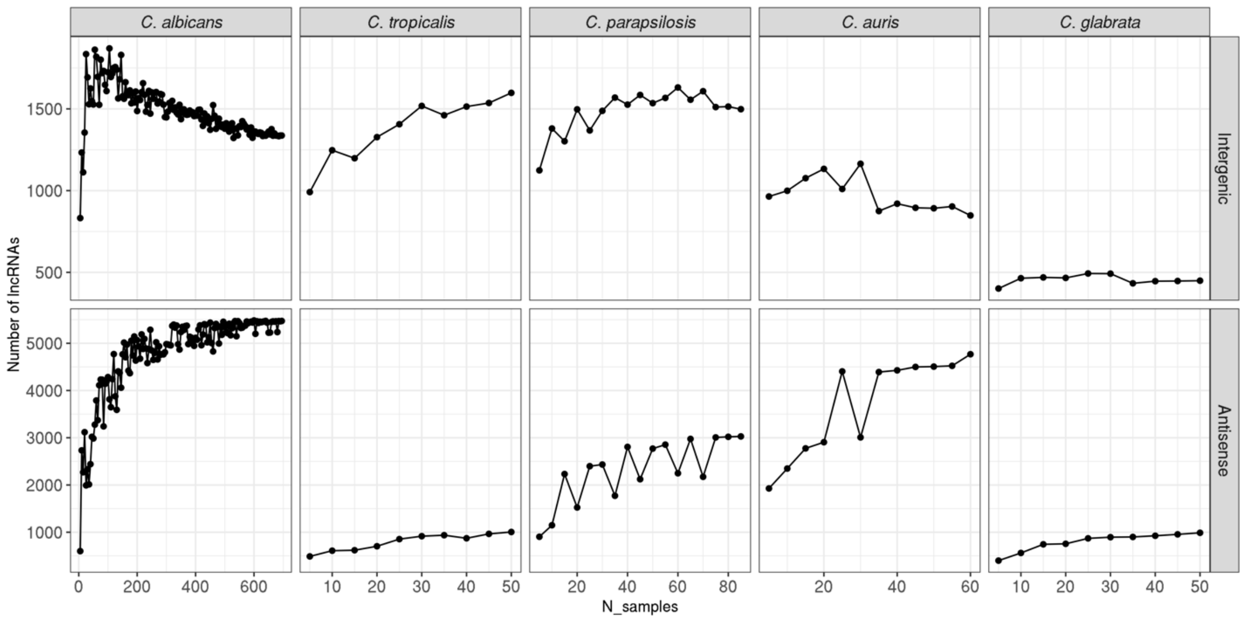

Figure S2: Saturation plots showing the number of identified lncRNAs depending on the number of analyzed samples. Per each species, samples were chosen randomly and the number of analyzed samples was incremented by 5 at each step.

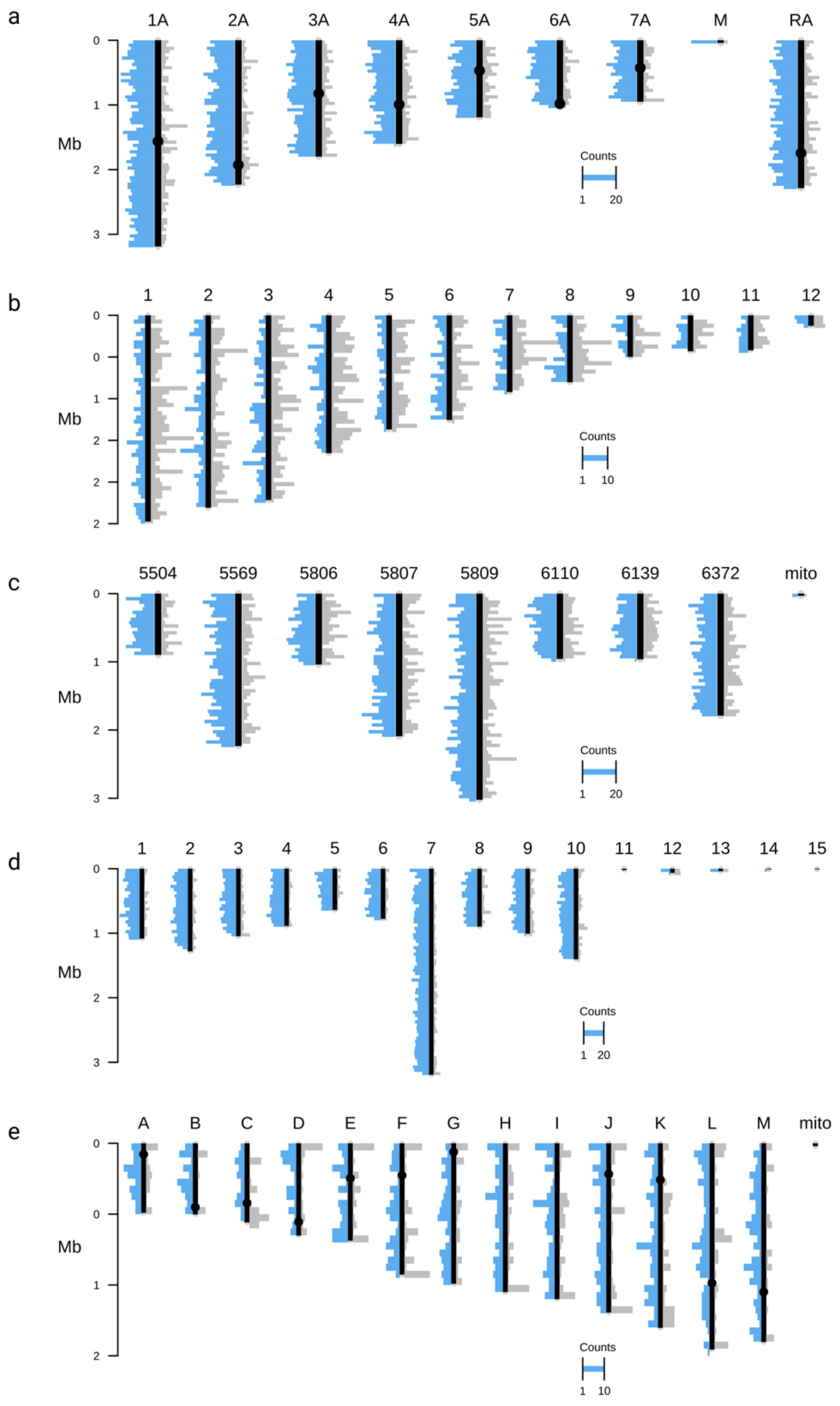

Figure S3: Distribution of lncRNAs across chromosomes of the studied species. Plots for **(a)** *C. albicans*; **(b)** *C. tropicalis*; **(c)** *C. parapsilosis,* **(d)** *C. auris* and **(e)** *C. glabrata*. The chromosomes are depicted with black vertical lines, centromeres (for *C. albicans* and *C. glabrata*) are depicted with black circles. The number of antisense transcripts is depicted in blue (at the left side of chromosomes), of intergenic ones - in grey (at the right side of chromosomes). Each bar represents a histogram of number of lncRNAs across 50kb-long windows. For *C. tropicalis*, contigs smaller than 100 kb are not shown (i.e. contigs 13-23).

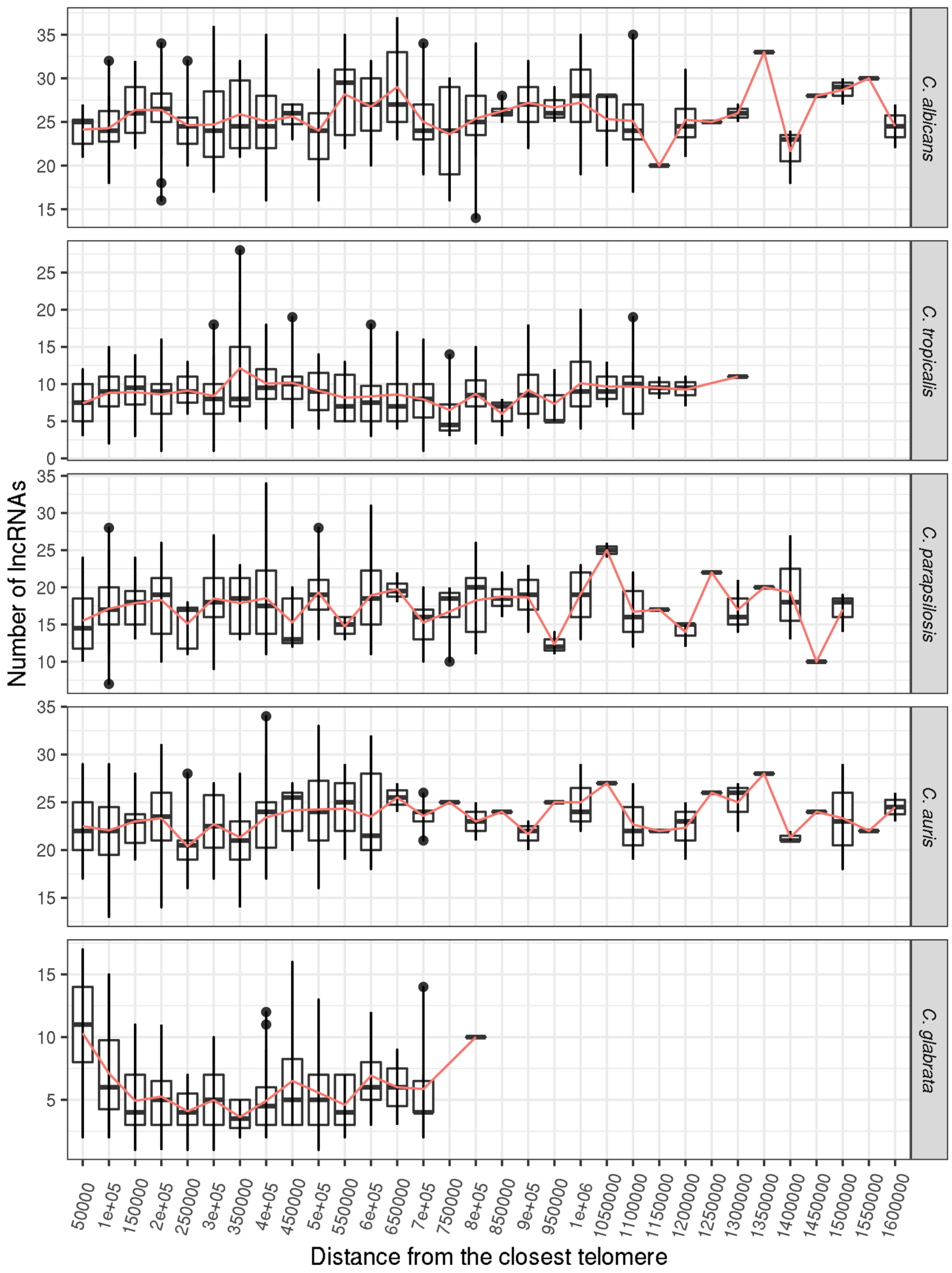

Figure S4: For each Candida species, the number of both intergenic and antisense lncRNAs (represented by bar plots) relative to the distance to the closest telomere across 50 kb windows. The data is summed across all chromosomes. The red line across each plot represents the mean.

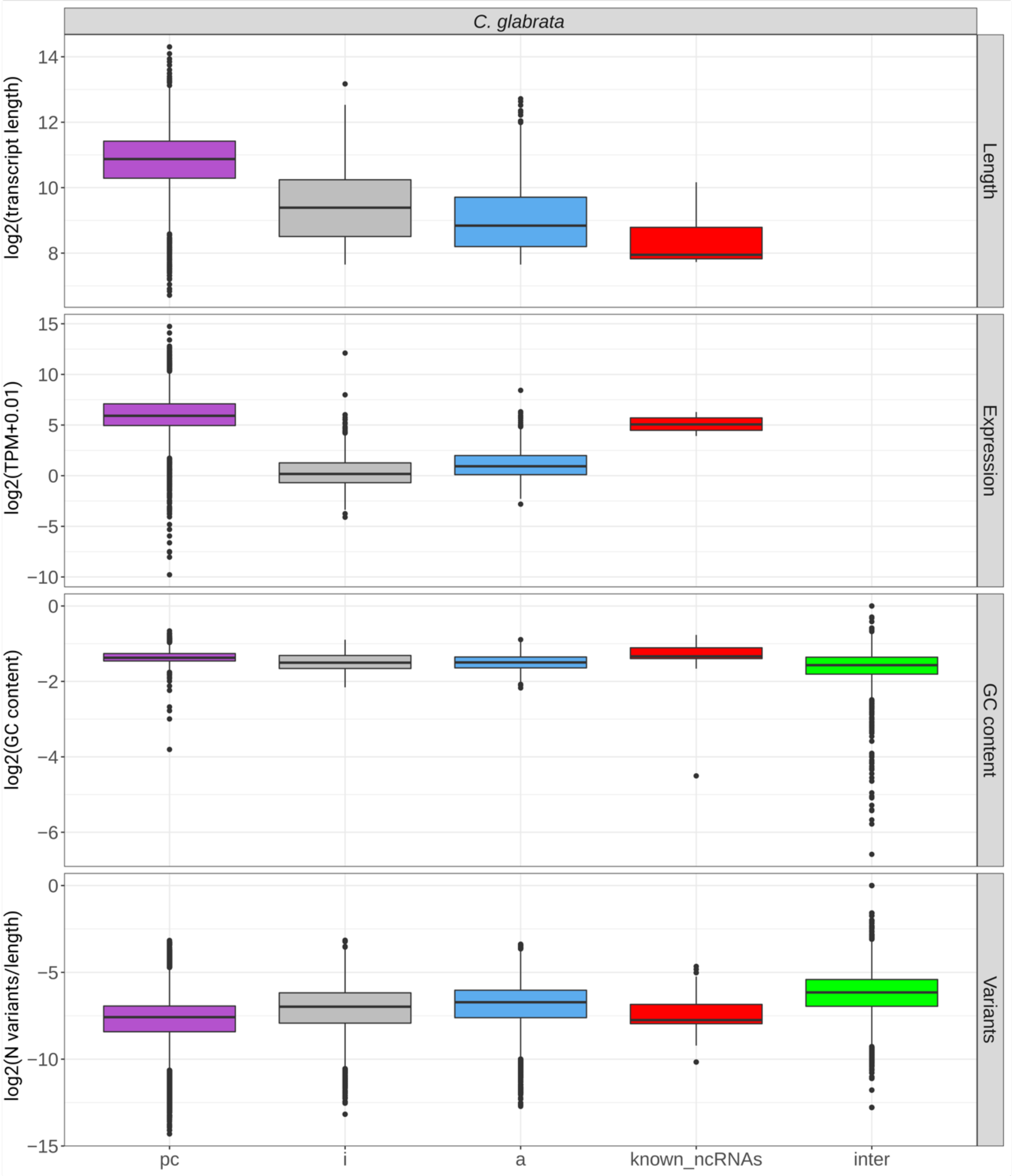

Figure S5: Comparison of different transcript properties of protein-coding genes (“pc”), lncRNAs found in the current study (“i” and “a” for intergenic and antisense transcripts, respectively), previously annotated lncRNAs (“known_ncRNAs”), and intergenic regions (“inter”) in *C. glabrata.*

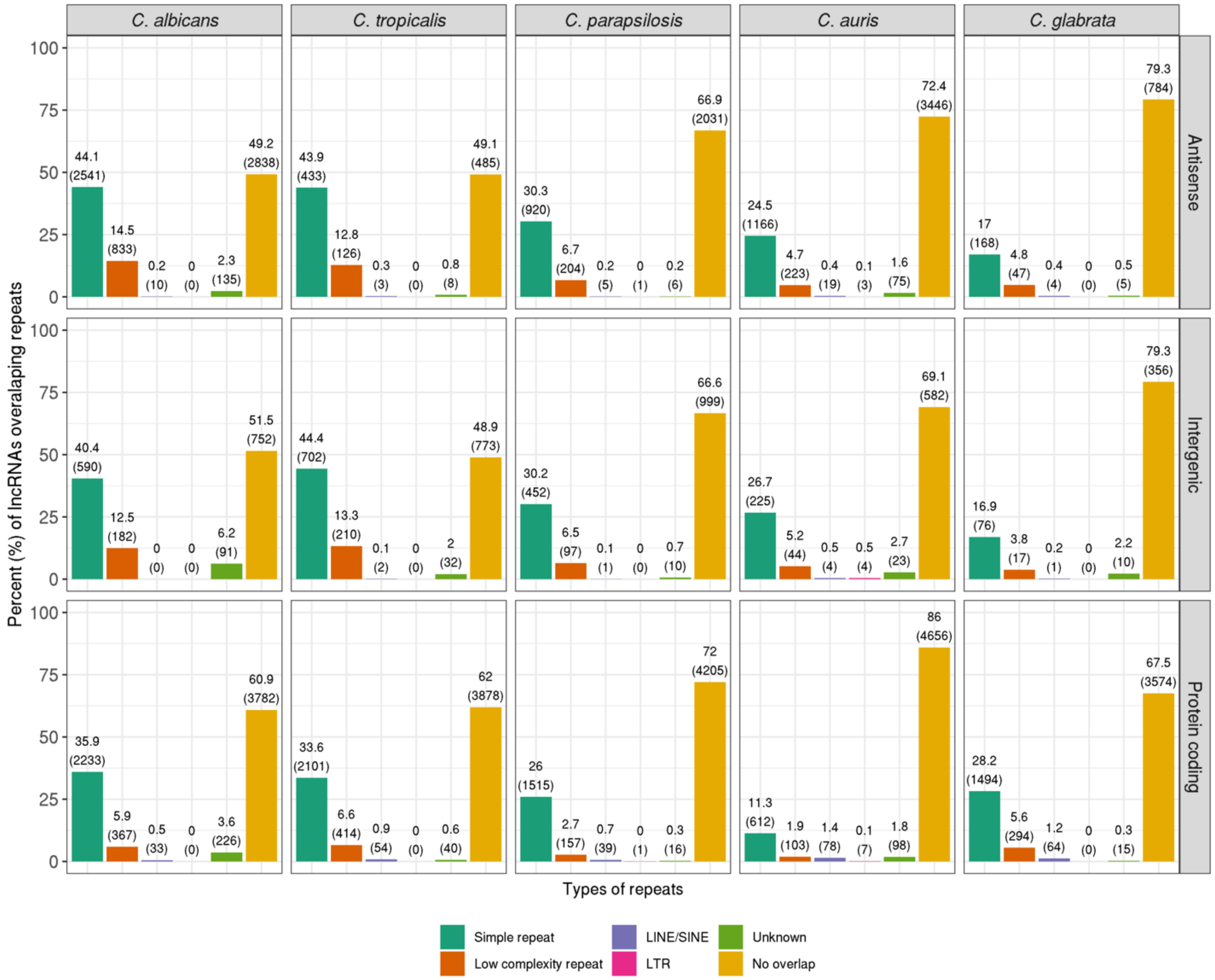

Figure S6: Percentage of lncRNAs overlapping repeat regions found in *Candida* genomes. Cumulative percentage for each species and lncRNA type can exceed 100% since one lncRNA can overlap more than one class of repeats. Numbers on the bar plot indicate the percentage (at the top) and number (at the bottom) of lncRNAs overlapping repeats.

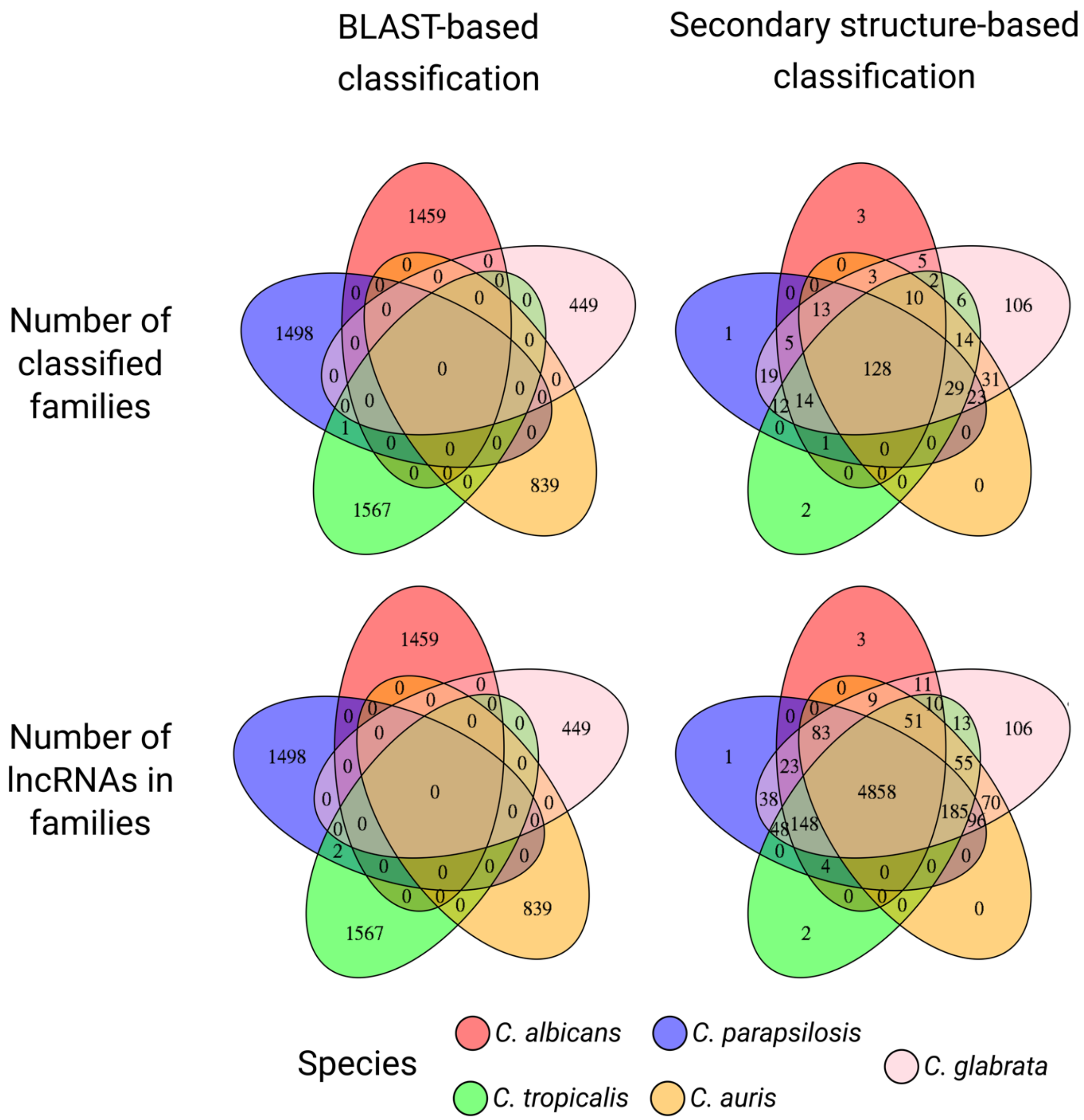

Figure S7: Assessment of evolutionary relationships of intergenic lncRNAs across *Candida* species. Each Venn diagram represents the number of classified lncRNAs families (top row) and the number of corresponding lncRNA in those families (bottom raw) across the species. The two used methodologies to generate the relatedness data corresponding to the Venn diagrams are mentioned at the top of the plot, namely BLAST-based classification on the left and secondary structure-based classification at the right.

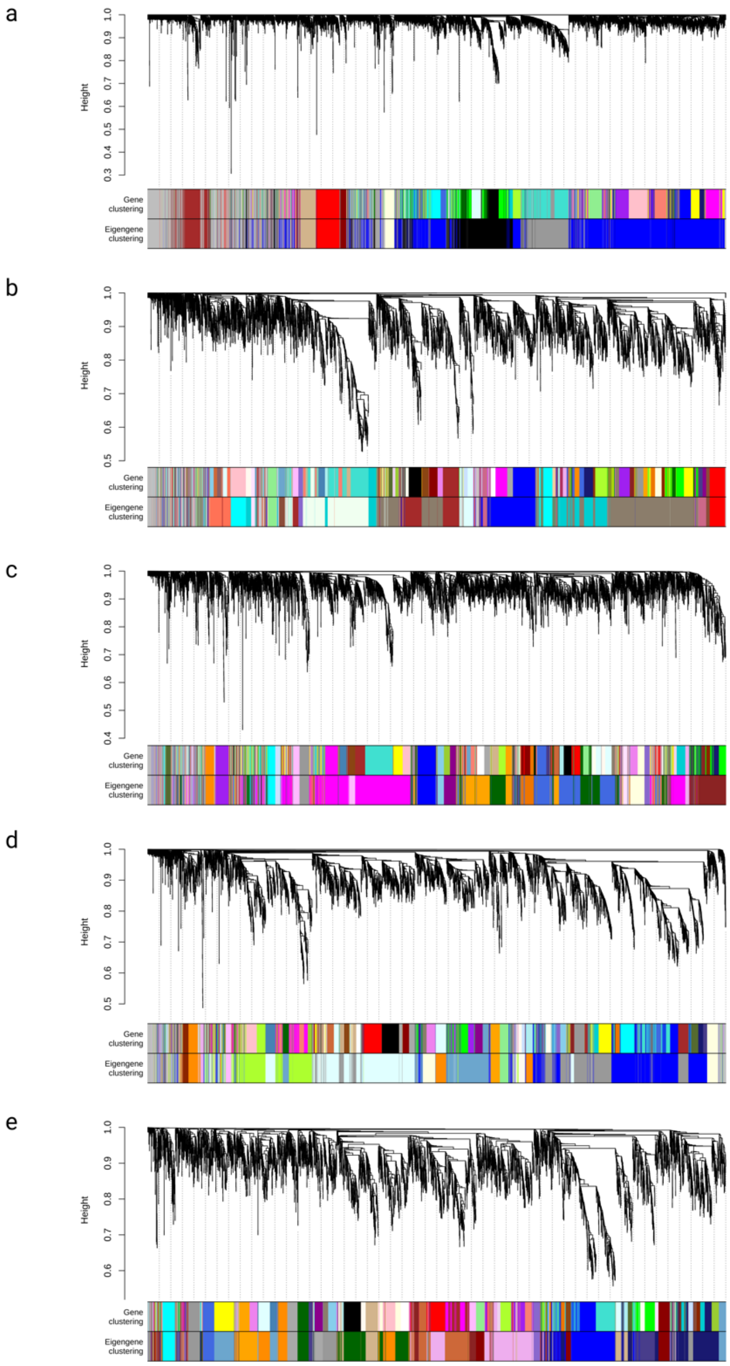

Figure S8: Gene co-expression networks represented as dendrograms produced with WGCNA and hclust function based on 1-Topology Overlap Matrix. Networks for (a) *C. albicans*; (b) *C. tropicalis*; (c) *C. parapsilosis*, (d) *C. auris* and (e) *C. glabrata*. Each dendrogram represents co-expressed modules (on the bottom) obtained by gene clustering (top row) and eigengene clustering (bottom row).

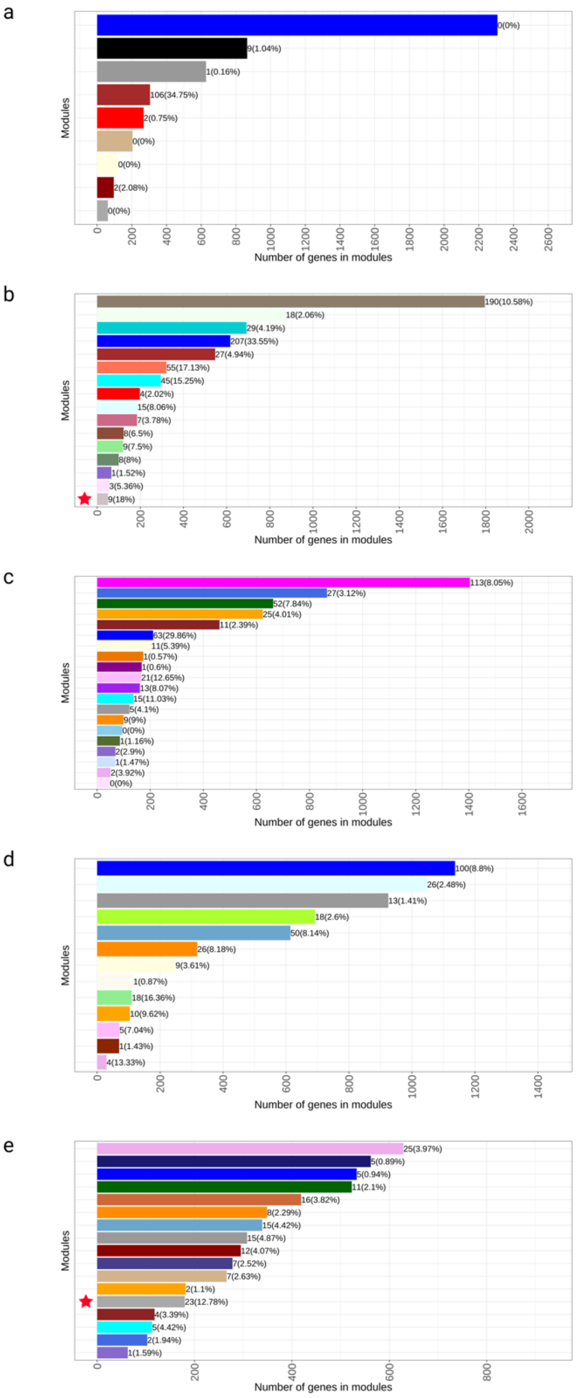

Figure S9: Co-expressed modules and distribution of lncRNAs in modules for studied *Candida* species. Modules for (a) *C. albicans;* (b) *C. tropicalis;* (c) *C. parapsilosis*; (d) *C. auris* and (e) *C. glabrata*. Each bar plot represents a module, which height corresponds to the number of genes (both protein-coding and intergenic lncRNAs) involved in the module. Numbers at the right of bars represent the number of lncRNAs in the module and proportion (in parentheses) of lncRNA over the total number of genes in the module. Modules where a lncRNA represents a hub is highlighted by a red star.

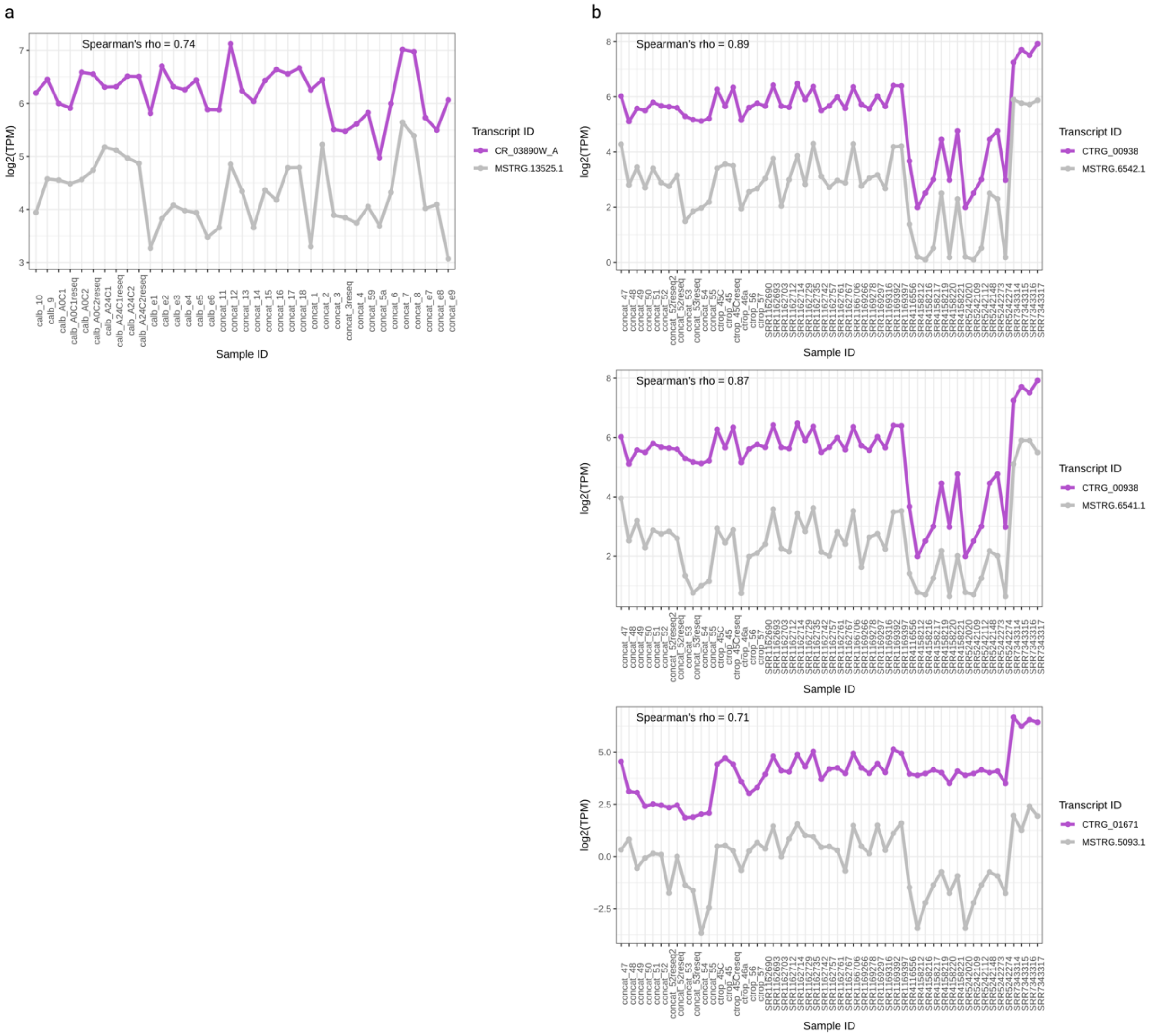

Figure S10: Expression levels of infection-specific lncRNAs and co-expressed protein coding genes across analyzed sample in (a) module "darkred" of *C. albicans* (for visualization purposes only samples from S dataset are shown) and (b) module coral1 of *C. tropicalis*. Only co-expression of infection-specific lncRNAs with highest WGCNA weights are shown. Sample IDs correspond to SRA identifiers and internal samples names in case of S dataset.

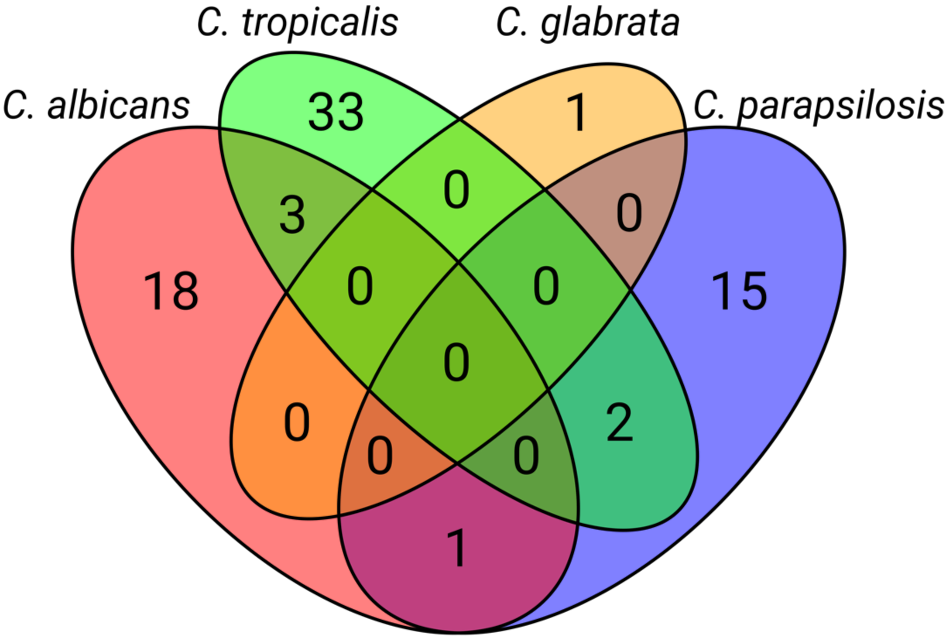

Figure S11: Venn diagram of syntenic families where “infection-specific” lncRNAs of the four *Candida* pathogens are involved in.

Supplementary Note 1: Comparison of transcriptome assemblies between Stringtie and Trinity software.

To assess the impact of our data analysis pipeline on the results of transcriptome assembly, we compared the transcriptome assemblies produced by Stringtie v. 1.3.3b of all analyzed samples with those produced by a popular *de novo* transcriptome assembler Trinity v. 2.8.5. This comparative analysis was based on the following steps.

All the samples that were used for final transcriptome reconstruction with Stringtie were also assembled with Trinity

For a fair comparison with Stringtie, and considering that high quality reference genomes for all the analyzed species are available (and also as suggested by Trinity developers https://github.com/trinityrnaseq/trinityrnaseq/wiki/Genome-Guided-Trinity-Transcriptome-Assembly), we have run Trinity v. 2.8.5 with the genome-guided mode (i.e. --genome_guided_bam) by supplying to it the same bam files used for Stringtie assembly. Additionally, we controlled for the maximum intron length (--genome_guided_max_intron 1000, similarly as we did on the TopHat2 mapping step) and strand-specificity of the data.

Generating gff file for both Trinity and Stringtie assemblies

Considering that Trinity produces a fasta file with assembled transcripts whereas Stringtie outputs a gff file, we converted Trinity fasta file to a gff file so we could further compare both assemblies with gffcompare tool, which apart from the sequence also provides other important information such as a class of a transcript, strand, location, etc. To do this, we mapped Trinity assemblies to the corresponding reference genomes using GMAP v. 2016-11-07 software[1], which produces a gff file of alignments. To avoid biases in the comparison due to this mapping step, we also performed this step with Stringtie assemblies. For this, we first generated a fasta file of transcripts from the corresponding reference genomes and gff files produced by Stringtie assembly using gffread v.0.12.1[2]. Then we also mapped this fasta file to the corresponding reference using GMAP and generated a gff file from the fasta sequences of Stringtie assembly. In this way, we had a gff file for both Stringtie and Trinity assemblies, which were generated by the same mapping software, thus ensuring fair downstream comparisons.

Assessment of sensitivity and specificity of both assemblers

Subsequently, we used the standard method of evaluating sensitivity and specificity of transcriptome reconstruction as described elsewhere [3–5]. We used gffcompare v0.11.2 software to compare assemblies of both software to the reference genome annotations,

allowing us to calculate sensitivity and specificity. Gffcompare assesses these two parameters as following:

$$Sensitivity = TP / M$$

$$Specificity = TP / N$$

TP - "true positives" - query features (i.e. assembled transcripts in this case) which agree with the corresponding reference annotation features;

M - total number of reference features;

N - total number of query features.

The results of this analysis are presented in Fig. S12. Stringtie produced assemblies with a very high rate of sensitivity (Fig. S12a) meaning that it almost does not miss any transcripts in the query data even if they are present in low abundances (which is specifically important in the context of lncRNAs). This result is somewhat expected since this software is a genome and annotation-guided assembler. In contrast, Trinity showed lower levels of sensitivity, indicating that the algorithm for de novo assembly can miss many annotated transcripts. One explanation for this result could be that de novo assembly heavily relies on high sequencing depth to produce reliable transcripts, while lowly expressed features can be missed [6,7].

For the specificity metrics (Fig. S12b), which indicates how many novel previously non-annotated transcripts are recovered in the query data, we observed that Stringtie shows overall higher values of specificity over Trinity, indicating that the latter assembles higher number novel transcripts.

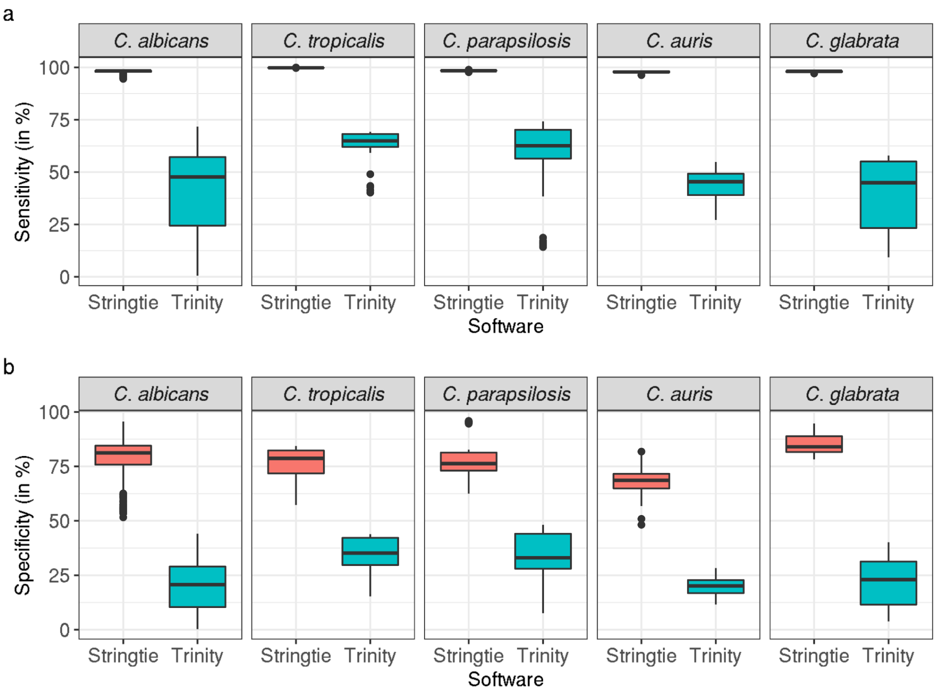

Figure S12: (a) Sensitivity and (b) specificity parameters of Stringtie and Trinity across studied yeast pathogens. Box plots are produced using sensitivity and specificity values of all samples used in the final transcriptome assemblies (*n*=699 for *C. albicans, n*=53 for *C. tropicalis*, *n*=86 for *C. parapsilosis*, *n*=61 for *C. auris* and *n*=51 for *C. glabrata*).

Assessment of assembly consistency between both software

Further analysis showed that overall Trinity assembled more transcripts than Stringtie (Fig. S13a). However, by calculating the number of uniquely assembled transcripts by each software (i.e. transcripts of a given software's assembly which do not overlap with any transcript of another software's assembly), which was done by using bedtools intersect reciprocally between two assemblies, we found that Stringtie assembles more of unique transcripts (Fig. S13b). Based on these data, two inferences can be proposed.

First, the fact that Trinity assembles more transcripts in total but has less of unique transcripts indicates that Trinity transcripts tend to be more fragmented and those fragments overlap with Stringtie's transcripts. This idea is also supported by the distribution of transcript class codes as reported by gffcompare (Fig. S13c, see Fig. S14 for the description of each class code). For example, while Stringtie successfully assembles more transcripts which coincide with annotated features (class code "="),

Trinity as a rule finds more of partially matching transcripts (class code “c”), indicating a higher rate of fragmentation.

Second, the higher number of unique transcripts assembled by Stringtie indicates that Trinity can miss transcripts as described above (also see examples below) likely due to low expression levels. Nevertheless, we found that overall the assemblies between Stringtie and Trinity were largely consistent based on the low percentage of uniquely assembled transcripts between each software (Fig. S13d). For example, the highest difference was observed in case of *C. albicans*, where Stringtie on average assembled 17,5% of unique transcripts (out of its total number of Stringtie transcripts) and Trinity - 3.7%, with the rest being consistent between the two software.

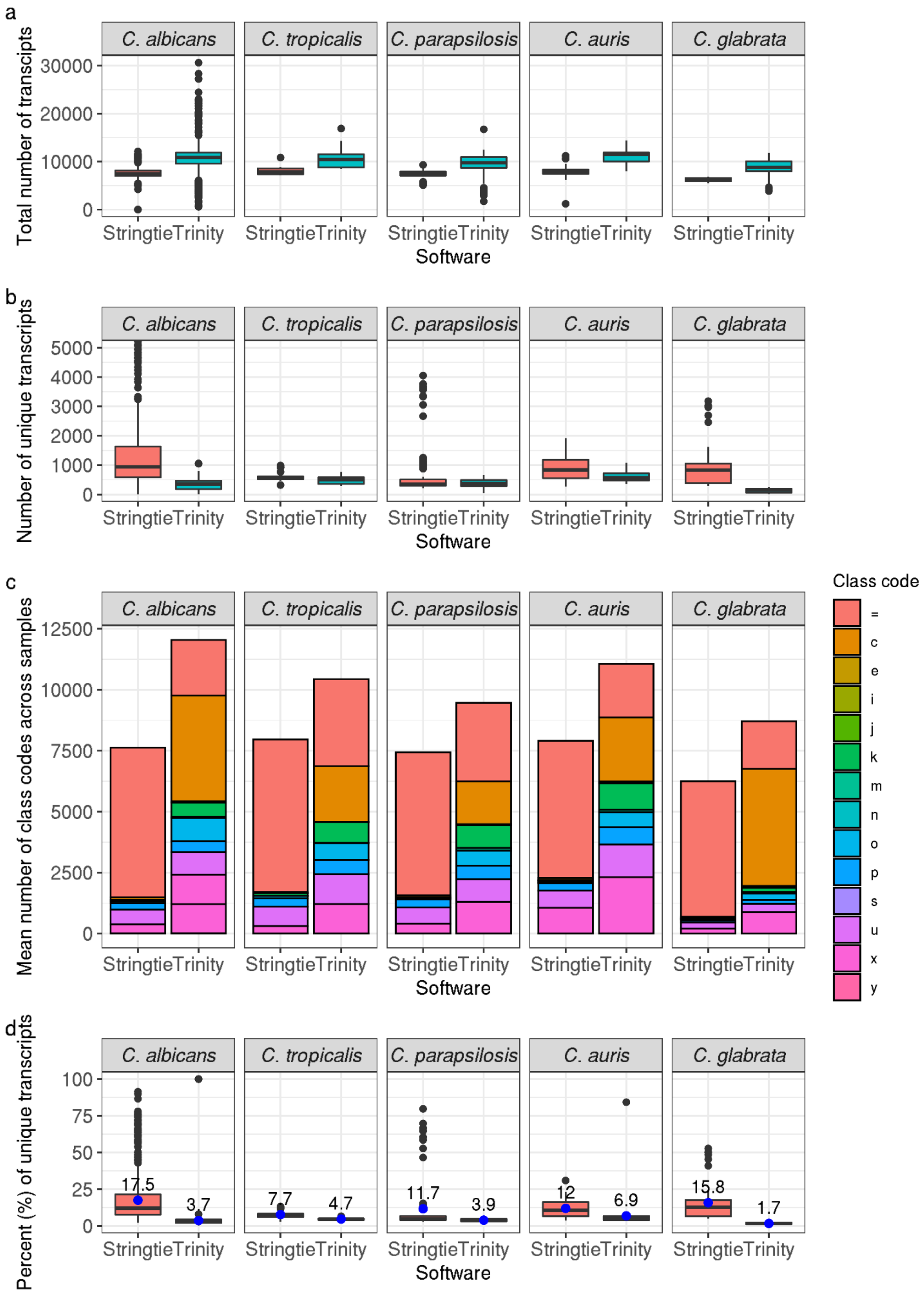

Figure S13: Comparison of transcriptome assemblies between Stringtie and Tiniry. (a) Total number of assembled transcripts; (b) Number of uniquely assembled transcripts by each software; (c) Distribution of gffcompare transcript class codes observed in

assemblies; and (d) Percentage of uniquely assembled transcripts by each software. Blue dots and corresponding numbers on top indicate mean values.

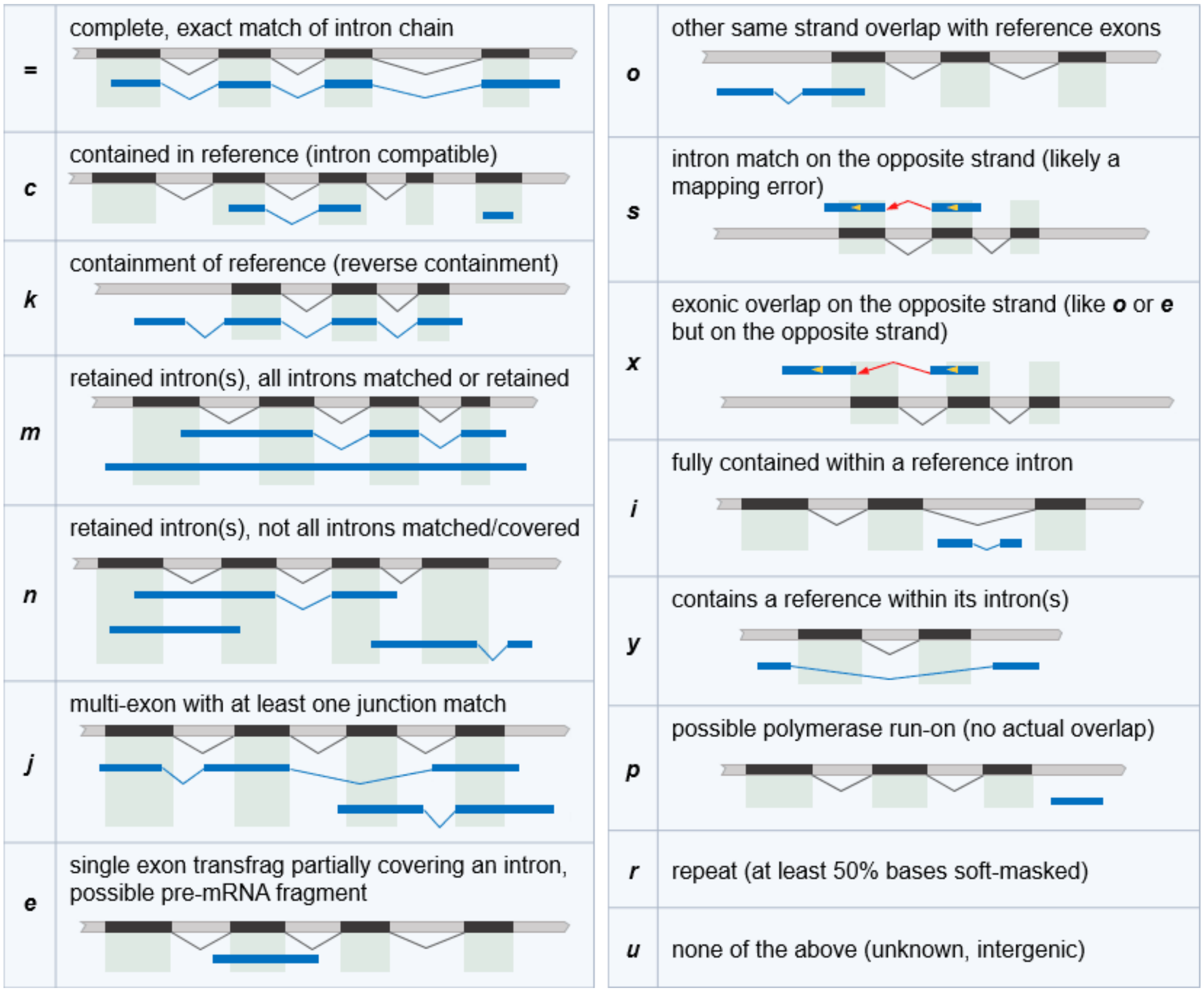

Figure S14: Description of gffcompare class codes. Image adopted from https://ccb.jhu.edu/software/stringtie/gffcompare.shtml

Assessing consistency between antisense and intergenic novel transcripts of the two Software

We then performed similar comparisons only for novel antisense and intergenic transcripts, which correspond to our lncRNAs (given the lack of their coding potential). As for the total number of transcripts, we observed that Trinity produces substantially more of both types of transcripts (especially antisense ones) compared to Stringtie (Fig. S15a).

However, in contrast to what was observed in the case of the total number of transcripts, Trinity also produces substantially more unique transcripts (Fig. S15b,c), demonstrating that Stringtie is more conservative for novel transcript discovery, specifically in the case of antisense transcripts, while there is a higher consensus between intergenic transcripts predicted by the two assemblers. Such a high rate of antisense transcript discovery by Trinity can be the result of several factors, including true biological variability but more likely the aforementioned transcript fragmentation, and strand confusion by Trinity as we show with some examples below. It must be noted, however, that discerning the relative impact of those factors in the observed high rate of antisense transcript discovery is problematic and would require extensive experimental testing specifically for the biological one.

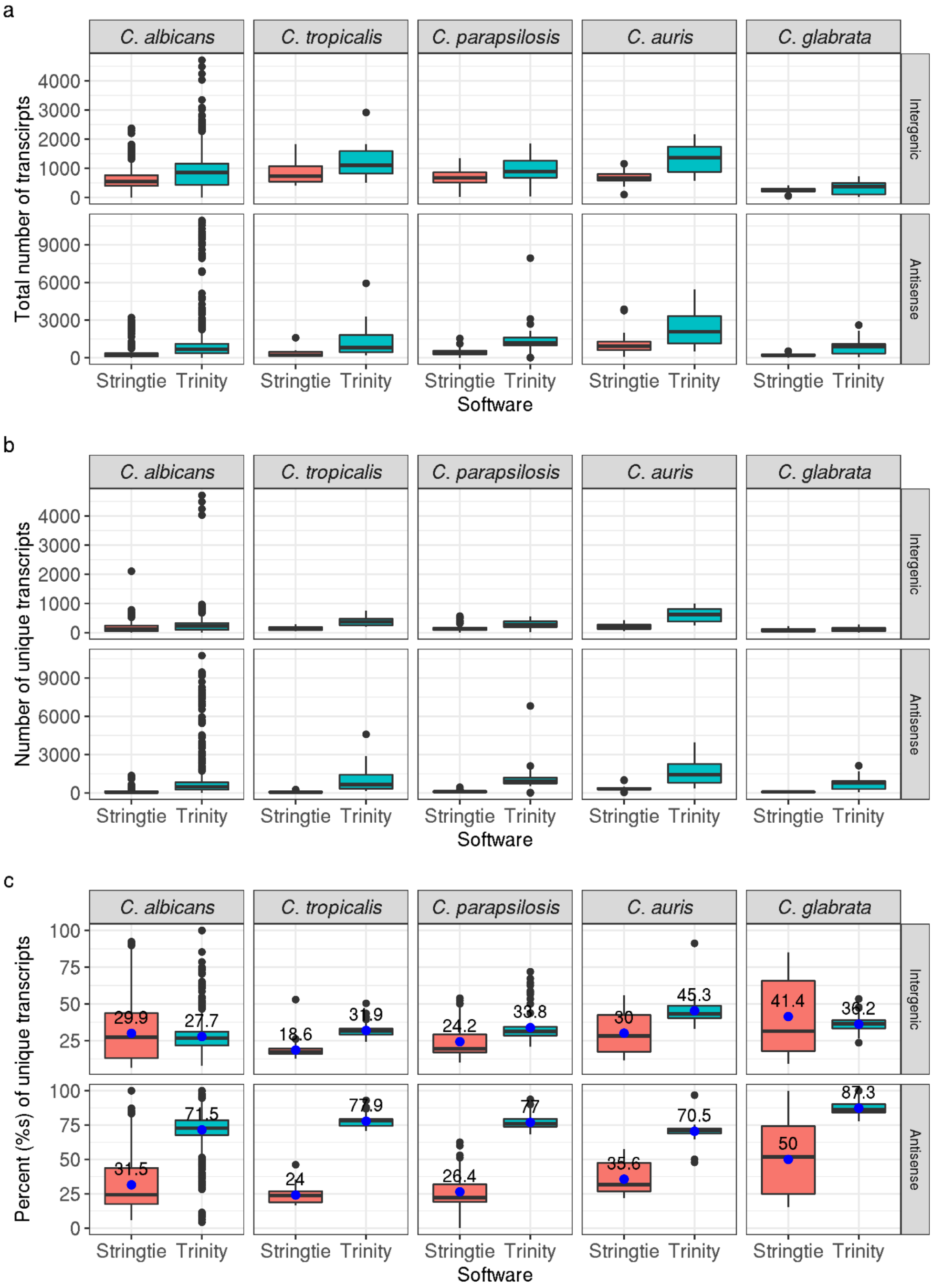

Figure S15: Comparison of transcript assemblies based on novel antisense and intergenic transcripts. (a). Total number of antisense and intergenic transcripts; (b) Number of unique antisense and intergenic transcripts; (c) Percentage of unique antisense and intergenic transcripts. Blue dots and corresponding numbers on top indicate mean values

Assessment of transcript reconstruction compared to independently experimentally identified lncRNA DINOR

We further compared Stringtie and Trinity assembly results with that of an independently identified lncRNA in *C. auris*. During the process of revision, Gao et al. (2021)[8] have reported a study where they identified a novel lncRNA named DINOR in *C. auris*, which is a virulence factor and global regulator of stress responses in this pathogen. Without having the sequencing data of that study, our analysis of *C. auris* public data using Stringtie also identified the lncRNA MSTRG.10503.1 corresponding to DINOR, i.e. located on the same chromosomal position (chromosome 9 between the genes B9J08_004840 and B9J08_004841, and overlapping the former) and the same strand

We then manually investigated assemblies of several randomly chosen *C. auris* samples using IGV v. 2.5.3 software [9] and checked whether or not Trinity also was able to identify that transcript (Fig. S16, for better visibility only sample SRR8351139 is shown). While Stringtie consistently reported DINOR, Trinity didn't identify it in all the tested samples. Moreover, a closer manual inspection of neighboring regions in Trinity assemblies showed some fused and/or fragmented transcripts, missing reference transcripts and transcripts with incorrect strand information (as discussed above).

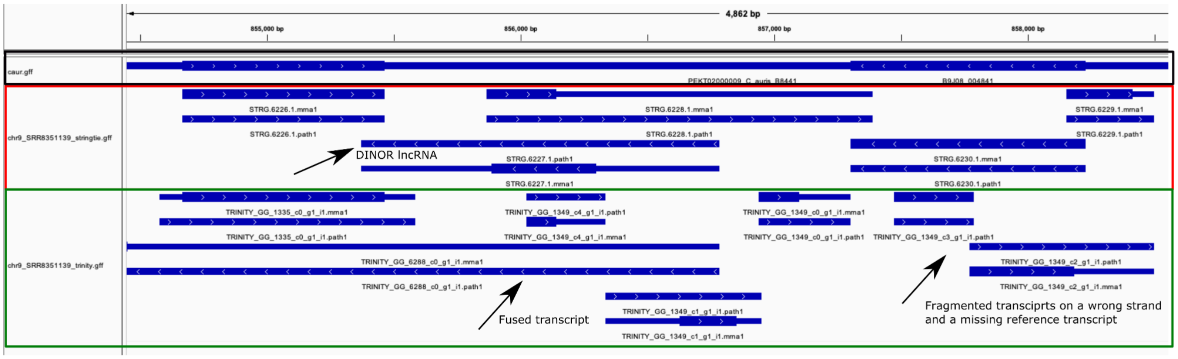

Figure S16: IGV screenshot of Stringtie (red box) and Trinity (green box) assembly compared to reference annotations (black box) in the region of DINOR lncRNA location (chromosome 9 between genes B9J08_004840 and B9J08_004841).

Based on the above described results, the genome-guided transcriptome assembly of Stringtie overall produced more complete and accurate results compared to those of *de novo* Trinity assembly. We thus used the results of Stringtie assemblies for all downstream analyses.

References

1. Wu, T. D. & Nacu, S. Fast and SNP-tolerant detection of complex variants and

splicing in short reads. *Bioinformatics* **26**, 873–881 (2010).

2. Pertea, G. & Pertea, M. GFF Utilities: GffRead and GffCompare. *F1000Res.* **9**, (2020).

3. You, B.-H., Yoon, S.-H. & Nam, J.-W. High-confidence coding and noncoding transcriptome maps. *Genome Res.* **27**, 1050–1062 (2017).

4. Tao, X. *et al.* Digital gene expression analysis based on integrated de novo transcriptome assembly of sweet potato [Ipomoea batatas (L.) Lam]. *PLoS One* **7**, e36234 (2012).

5. Song, L., Sabunciyan, S., Yang, G. & Florea, L. A multi-sample approach increases the accuracy of transcript assembly. *Nat. Commun.* **10**, 5000 (2019).

6. Chow, K.-S., Ghazali, A.-K., Hoh, C.-C. & Mohd-Zainuddin, Z. RNA sequencing read depth requirement for optimal transcriptome coverage in Hevea brasiliensis. *BMC Res. Notes* **7**, 69 (2014).

7. Li, F.-D., Tong, W., Xia, E.-H. & Wei, C.-L. Optimized sequencing depth and de novo assembler for deeply reconstructing the transcriptome of the tea plant, an economically important plant species. *BMC Bioinformatics* **20**, 553 (2019).

8. Gao, J. *et al.* LncRNA DINOR is a virulence factor and global regulator of stress responses in Candida auris. *Nat Microbiol* **6**, 842–851 (2021).

9. Robinson, J. T. *et al.* Integrative genomics viewer. *Nat. Biotechnol.* **29**, 24–26 (2011).
