# Supplementary material for: The long non-coding RNA landscape of Candida yeast pathogens
Source: Nat Commun. 2021 Dec 16;12:7317. doi: 10.1038/s41467-021-27635-4 (PMC8677757; doi:10.1038/s41467-021-27635-4)
Supplement: Supplementary file 2 — Description of Additional Supplementary Files [file 41467_2021_27635_MOESM2_ESM.pdf]

Description of Additional Supplementary Files

File Name: Supplementary Data 1
Description: All RNA-Seq datasets used in this study;

File Name: Supplementary Data 2
Description: lncRNA catalogues of the studied species. For lncRNA type, x - antisense lncRNAs, u - intergenic lncRNAs;

File Name: Supplementary Data 3
Description: Mean values of different parameters of lncRNAs, protein coding genes and intergenic regions (where applicable). Within each species, values highlighted with black boxes are not significantly different from each other (e.g. mean length of intergenic and antisense lncRNAs in C. auris; see the main text for details);

File Name: Supplementary Data 4
Description: Pairwise syntenic information between lncRNAs across studied species;

File Name: Supplementary Data 5
Description: Influence of synteny classification parameters (see Materials and Methods for details) on the obtained number of syntenic families;

File Name: Supplementary Data 6
Description: Influence of synteny classification parameters (see Materials and Methods for details) on the obtained number of syntenic lncRNAs;

File Name: Supplementary Data 7
Description: Co-expressed modules of lncRNAs and protein-coding genes, their content and GO term, PFAM and KEGG enrichments. Only 5 enrichments with lowest p-values are shown;

File Name: Supplementary Data 8
Description: Results of differential expression analysis of *C. albicans* lncRNAs during the course of epithelial cell infection;

File Name: Supplementary Data 9
Description: Results of differential expression analysis of *C. tropicalis* lncRNAs during the course of epithelial cell infection;

File Name: Supplementary Data 10
Description: Results of differential expression analysis of *C. parapsilosis* lncRNAs during the course of epithelial cell infection;

File Name: Supplementary Data 11
Description: Results of differential expression analysis of *C. glabrata* lncRNAs during the course of epithelial cell infection;

File Name: Supplementary Data 12
Description: Results of running BLASTn of lncRNAs against each fungal species genome;

File Name: Supplementary Data 13
Description: Results of running BLASTn of lncRNAs against each fungal species genome. Numbers in the matrix indicate the number of lncRNAs with significant hits, and the number of significant hits in parentheses;

File Name: Supplementary Data 14
Description: List of lncRNAs which are putative tRNA/ncRNA and thus are discarded from further analysis.
