# Supplementary material for: The long non-coding RNA landscape of Candida yeast pathogens
Source: Nat Commun. 2021 Dec 16;12:7317. doi: 10.1038/s41467-021-27635-4 (PMC8677757; doi:10.1038/s41467-021-27635-4)
Supplement: Supplementary file 17 — Reporting Summary [file 41467_2021_27635_MOESM17_ESM.pdf]

nature portfolio

Corresponding author(s): Toni Gabaldón

Last updated by author(s): 18 November 2021

Reporting Summary

Nature Portfolio wishes to improve the reproducibility of the work that we publish. This form provides structure for consistency and transparency in reporting. For further information on Nature Portfolio policies, see our Editorial Policies and the Editorial Policy Checklist.

Statistics

For all statistical analyses, confirm that the following items are present in the figure legend, table legend, main text, or Methods section.

n/a	Confirmed	
☐	☒	The exact sample size (n) for each experimental group/condition, given as a discrete number and unit of measurement
☒	☐	A statement on whether measurements were taken from distinct samples or whether the same sample was measured repeatedly
☐	☒	The statistical test(s) used AND whether they are one- or two-sided<br>*Only common tests should be described solely by name; describe more complex techniques in the Methods section.*
☐	☒	A description of all covariates tested
☐	☒	A description of any assumptions or corrections, such as tests of normality and adjustment for multiple comparisons
☐	☒	A full description of the statistical parameters including central tendency (e.g. means) or other basic estimates (e.g. regression coefficient) AND variation (e.g. standard deviation) or associated estimates of uncertainty (e.g. confidence intervals)
☐	☒	For null hypothesis testing, the test statistic (e.g. F, t, r) with confidence intervals, effect sizes, degrees of freedom and P value noted<br>*Give P values as exact values whenever suitable.*
☒	☐	For Bayesian analysis, information on the choice of priors and Markov chain Monte Carlo settings
☒	☐	For hierarchical and complex designs, identification of the appropriate level for tests and full reporting of outcomes
☒	☐	Estimates of effect sizes (e.g. Cohen's d, Pearson's r), indicating how they were calculated

Our web collection on statistics for biologists contains articles on many of the points above.

Software and code

Policy information about availability of computer code

Data collection	sratoolkit v. 2.9.6-1
Data analysis	FastQC v. 0.11.6<br>Multiqc v. 1.0<br>Trimmomatic v. 0.36<br>RSEM prepare-reference v. 1.3<br>salmon v. 0.8.1<br>TopHat2 v. 2.1.1<br>samtools v. 1.3.1<br>Stringtie v. 1.3.3b<br>Trinity v. 2.8.5<br>gffcompare v. 0.11.2<br>CGAT gtf2gtf v.0.3.2<br>CPC v. 0.9<br>Feelnc v. 0.1.1<br>BLASTn v.2.9<br>Featurecounts v. 1.6.4<br>chromPlot v. 1.14.0<br>bedtools v.2.29.2<br>RepeatModeler v. 2.0.1<br>RepeatMasker v. 4.0.9<br>perSVade v. 0.10<br>Synima pipeline (downloaded on August 1st, 2021)

RNAfold v. 2.4.14
Beagle v.0.2
MEME suit v. 4.11.2
lncLOOM v. 1.0
WGCNA v. 1.69
clusterProfiler v. 3.14.3
R v. 3.6.1
Cytoscape v. 3.7.2
DESeq2 v. 1.26.0
RUV v. 1.20.0
IGV v. 2.5.3
The whole data analysis pipeline, i.e. all scripts, datasets, annotations, software versions and auxiliary files to reproduce the results of the study, plots and supplementary materials, are available at our GitHub page https://github.com/Gabaldonlab/lncRNAs. Any updates to the codes and supplementary materials will be available in our GitHub page.

For manuscripts utilizing custom algorithms or software that are central to the research but not yet described in published literature, software must be made available to editors and reviewers. We strongly encourage code deposition in a community repository (e.g. GitHub). See the Nature Portfolio guidelines for submitting code & software for further information.

Data

Policy information about availability of data

All manuscripts must include a data availability statement. This statement should provide the following information, where applicable:

- Accession codes, unique identifiers, or web links for publicly available datasets
- A description of any restrictions on data availability
- For clinical datasets or third party data, please ensure that the statement adheres to our policy

All publicly available RNA-Seq datasets were retrieved from NCBI SRA database (see Supplementary Data 1 for details of all samples used in this study). Reference genomes, genome annotations, orthology information, GO and PFAM domain tables were obtained from Candida Genome Database. Variant calling data were obtained from Candidamine database. See “Methods” section for more details. All datasets generated in this study are available at our GitHub page https://github.com/Gabaldonlab/lncRNAs.

Field-specific reporting

Please select the one below that is the best fit for your research. If you are not sure, read the appropriate sections before making your selection.

☒ Life sciences ☐ Behavioural & social sciences ☐ Ecological, evolutionary & environmental sciences

For a reference copy of the document with all sections, see nature.com/documents/nr-reporting-summary-flat.pdf

Life sciences study design

All studies must disclose on these points even when the disclosure is negative.

Sample size	In our work we used all publicly available RNA-Seq datasets of the studied species from NCBI SRA database as of 19th of July 2019.
Data exclusions	Some RNA-Seq datasets were excluded due to 1) poor quality read data (based on Phred Scores), 2) unstrandedness of RNA-Seq data and 3) short read length <49 bp. In the latter case, we excluded data with very short reads since they can be problematic for transcriptome assembly step. See Supplementary Data 1 for details.
Replication	Replicates are not relevant for this study since 1) we did not perform any experiments, 2) our approach reconstructs a single consensus transcriptome per species across all analyzed samples.
Randomization	Randomization is not relevant for this study since we did not perform any experiments.
Blinding	Blinding is not relevant for this study since we did not perform any experiments.

Reporting for specific materials, systems and methods

We require information from authors about some types of materials, experimental systems and methods used in many studies. Here, indicate whether each material, system or method listed is relevant to your study. If you are not sure if a list item applies to your research, read the appropriate section before selecting a response.

Materials & experimental systems

n/a	Involved in the study
☒	☐ Antibodies
☒	☐ Eukaryotic cell lines
☒	☐ Palaeontology and archaeology
☒	☐ Animals and other organisms
☒	☐ Human research participants
☒	☐ Clinical data
☒	☐ Dual use research of concern

Methods

n/a	Involved in the study
☒	☐ ChIP-seq
☒	☐ Flow cytometry
☒	☐ MRI-based neuroimaging
